# OncomiR miR-182-5p Enhances Radiosensitivity by Inhibiting the Radiation-Induced Antioxidant Effect through SESN2 in Head and Neck Cancer

**DOI:** 10.3390/antiox10111808

**Published:** 2021-11-14

**Authors:** Min-Ying Lin, Yu-Chan Chang, Shan-Ying Wang, Muh-Hwa Yang, Chih-Hsien Chang, Michael Hsiao, Richard N. Kitsis, Yi-Jang Lee

**Affiliations:** 1 Department of Biomedical Imaging and Radiological Sciences, National Yang Ming Chiao Tung University, Taipei Branch, Taipei 112, Taiwan; milo841120@gmail.com (M.-Y.L.); yuchanchang@ym.edu.tw (Y.-C.C.); sywang@mail.femh.org.tw (S.-Y.W.); chchang@iner.gov.tw (C.-H.C.); 2 Department of Nuclear Medicine, Far Eastern Memorial Hospital, New Taipei City 220, Taiwan; 3 Institute of Clinical Medicine, National Yang Ming Chiao Tung University, Taipei Branch, Taipei 112, Taiwan; mhyangymu@gmail.com; 4 Division of Medical Oncology, Department of Oncology, Taipei Veterans General Hospital, Taipei 112, Taiwan; 5 Cancer Progression Research Center, National Yang Ming Chiao Tung University, Taipei Branch, Taipei 112, Taiwan; 6 Isotope Application Division, Institute of Nuclear Energy Research, Taoyuan 325207, Taiwan; 7 Genomics Research Center, Academia Sinica, Taipei 11529, Taiwan; mhsiao@gate.sinica.edu.tw; 8 Departments of Medicine (Cardiology) and Cell Biology and Wilf Family Cardiovascular Research Institute, Albert Einstein College of Medicine, New York, NY 10461, USA; richard.kitsis@einsteinmed.org

**Keywords:** miR-182/96/183 cluster, miR-182-5p, head and neck squamous cell carcinoma, radioresistance, antioxidant, SESN2

## Abstract

Radiotherapy is routinely used for the treatment of head and neck squamous cell carcinoma (HNSCC). However, the therapeutic efficacy is usually reduced by acquired radioresistance and locoregional recurrence. In this study, The Cancer Genome Atlas (TCGA) analysis showed that radiotherapy upregulated the miR-182/96/183 cluster and that miR-182 was the most significantly upregulated. Overexpression of miR-182-5p enhanced the radiosensitivity of HNSCC cells by increasing intracellular reactive oxygen species (ROS) levels, suggesting that expression of the miR-182 family is beneficial for radiotherapy. By intersecting the gene targeting results from three microRNA target prediction databases, we noticed that sestrin2 (SESN2), a molecule resistant to oxidative stress, was involved in 91 genes predicted in all three databases to be directly recognized by miR-182-5p. Knockdown of SESN2 enhanced radiation-induced ROS and cytotoxicity in HNSCC cells. In addition, the radiation-induced expression of SESN2 was repressed by overexpression of miR-182-5p. Reciprocal expression of the miR-182-5p and SESN2 genes was also analyzed in the TCGA database, and a high expression of miR-182-5p combined with a low expression of SESN2 was associated with a better survival rate in patients receiving radiotherapy. Taken together, the current data suggest that miR-182-5p may regulate radiation-induced antioxidant effects and mediate the efficacy of radiotherapy.

## 1. Introduction

Worldwide, head and neck squamous cell carcinoma (HNSCC) was responsible for 890,000 cases and 450,000 deaths in 2018, and these numbers are expected to increase by approximately one million new cases annually by 2030 [1,2]. HNSCC is ranked as the sixth most common human cancer in a cross-country study [3]. HNSCC is notorious for its high recurrence rate and local invasion, which leads to its high mortality [4,5]. Although adjuvant radiotherapy and chemotherapy are the primary strategies for the treatment of HNSCC, the development of radioresistance has been reported to be accompanied by tumor recurrence and local invasion after radiotherapy [6,7,8]. However, the underlying mechanisms of radiation-induced radioresistance in HNSCC remain to be investigated.

Radiotherapy can cause irreversible genetic changes as well as epigenetic modifications. In addition to the methylation status, it also contains the expression levels of various noncoding RNAs [9]. Endogenous small noncoding RNAs, called microRNAs (miRNAs), are processed from pri-miRNAs transcribed by RNA polymerase II [10]. The number of total human mature miRNAs is approximately 2300, and ~50% of these microRNAs have been annotated in miRBase V22 [11]. It is well known that microRNAs can imperfectly match conserved sequences of 3′-untranslated regions (3’UTRs) of various mature mRNAs and lead to translational suppression and/or mRNA degradation [12,13]. Several lines of evidence have demonstrated that environmental stresses, such as radiation, can disrupt the intracellular redox status and regulate different species of microRNAs via specific transcription factors, such as p53, nuclear factor κB (NFκB), hypoxia-inducible factor-1α (HIF-1α), c-Myc, and nuclear factor-erythroid 2 related factor 2 (Nrf2) [14,15,16,17,18,19,20,21]. On the other hand, miRNAs also target genes involved in antioxidant signaling to modulate ROS generation and DNA repair machinery [22,23,24]. MiR-182-5p, a member involved in the miR-183/96/182 cluster, potentially regulates more than 1000 genes and has a dual function in tumorigenesis [25]. Recent reports have demonstrated that miR-182-5p can protect cells against oxidative stress-induced apoptosis and damage in an atherosclerosis (AS) model and human lens epithelial cells [26,27]. However, the role of miR-182-5p in the regulation of tumor redox status, especially after radiotherapy, remains unclear. As overexpression of miR-182-5p is commonly found in human cancers [28,29,30,31,32,33], it is of interest to investigate whether miR-182-5p also affects oxidative stress, as has been shown in nontumor cells.

Sestrin2 (SESN2) is a modulator of redox status in eukaryotic cells. In mammals, SESN2 is a member of the highly conserved *PA26* gene family encoding the SESN1-3 proteins [34]. SESN2 has been confirmed to be induced by oxidative stress but is regarded as an antioxidant that compromises resultant cell damage [35,36,37,38]. However, SESN2 does not contain significant catalytic domains to diminish oxidative stress. Instead, it can promote various antioxidant signaling pathways, including Nrf2 activation and peroxiredoxin (Prx) recycling, to suppress ROS [39,40]. Oxidative stress-induced SESN2 expression is strongly dependent on the activation of the Nrf2 transcription factor and the c-Jun N-terminal kinase (JNK)/AP-1 pathway, as Nrf2 and AP-1 binding sequences have been found in the promoter region of the *SESN2* gene [41,42]. Additionally, it has been reported that arsenic trioxide (ATO)-induced oxidative stress can upregulate SESN2 via suppression of miR-182-5p [43]. The antioxidant role of SESN2 has been reported to be important for releasing oxidative stress in cardiovascular diseases, lipotoxicity of the liver, immune- and inflammation-related diseases, and multiorgan diseases [44,45,46,47]. Nevertheless, the role of SESN2 in human cancer remains to be investigated.

In this study, we found that the miR-182/96/183 cluster was up-regulated in HNSCC patients treated with the radiotherapy by the TCGA analysis. MiR-182-5p could directly target SESN2 mRNA, which was associated with up-regulation of ROS and enhanced radiosensitivity to ionizing radiation in HNSCC. The role of the miR-182-5p/SESN2 regulatory pathway on radiation responses, and clinical relevance, were discussed.

## 2. Materials and Methods

### 2.1. In Silico Database Analysis

The clinical information and genomic matrix file of The Cancer Genome Atlas (TCGA) database were downloaded from the OncoMir Cancer Database (OMCD, https://www.oncomir.umn.edu/omcd/ (accessed on 13 August 2020) and the USCS Xena browser website (https://xenabrowser.net/heatmap/ (accessed on 27 October 2020). For the assessment of gene expression and overall survival using publicly available datasets, the Kaplan–Meier method with the log-rank test was used to analyze the association of miRNAs and patient survival rates using the online KM plotter with public datasets. The genes predicted to be targeted by miR-182-5p in three microRNA prediction databases (PITA, miRanda, and TargetScan) were summarized using The Encyclopedia of RNA Interactomes (ENCORI) platform [48].

### 2.2. Cell Cultures

Human tongue squamous cell carcinoma SAS cells (National Yang Ming Chiao Tung University, Taipei, Taiwan) were provided by Prof. Muh-Hwa Yang and were cultured in DMEM (Life Technologies Inc., Carlsbad, CA, USA). Human hypopharyngeal carcinoma FaDu cells (American Type Culture Collection, Manassas, VA, USA) were maintained in RPMI-1640 (Life Technologies Inc., Carlsbad, CA, USA). To produce lentiviral particles, human embryonic kidney 293T cells (CRL-3216, American Type Culture Collection, Manassas, VA, USA) were cultured in DMEM. All cell lines were supplemented with 10% fetal bovine serum (Thermo Fisher Scientific Inc., Waltham, MA, USA), 2 mM L-glutamine, and 50 U/mL penicillin. Cells were incubated at 37 °C in a humidified incubator containing 5% CO_2_.

### 2.3. Gene Construction and Lentivirus Production

The pGIPZ-mir-182-5p lentiviral shRNAmir construct was purchased from Thermo Fisher (Thermo Fisher Scientific Inc., Waltham, MA, USA). This construct was cotransfected with pMDG and pCMV-Δ8.91 plasmids into 293T cells using the calcium phosphate transfection method. After 48 h of incubation, the supernatant was collected for cell infection in the presence of polybrene (8 µg/mL). The infected cells were then selected with puromycin (0.5 µg/mL) for one week.

### 2.4. Total RNA Extraction and qPCR Analysis

Total RNA was extracted using TRIzol reagent (Thermo Fisher Scientific Inc., Waltham, MA, USA) and then purified using a QIAGEN RNA mini kit (Thermo Fisher Scientific Inc., Waltham, MA, USA) following the manufacturer’s instructions. Extracted RNA was quantified using a NanoDrop Spectrophotometer (Thermo Fisher Scientific Inc., Waltham, MA, USA). Reverse transcription of mRNA was carried out using oligo dT primers and SuperScript III reverse transcriptase (Life Technologies Inc., Carlsbad, CA, USA). For microRNAs, stem loop-specific primers were used (Table 1). Quantitative polymerase chain reaction (qPCR) was performed using SYBR FAST Reagent (Kapa Biosystems Inc., Woburn, MA, USA), and the results were analyzed by StepOne Plus machine bundled software (Allied Business Intelligence Inc., Oyster Bay, NY, USA). Expression of the target gene was normalized to β-actin and U6 snRNA as experimental needs and endogenous controls using the ΔΔCt method.

### 2.5. Analysis of Reactive Oxygen Species (ROS)

Intracellular ROS were detected using 2,7-dichlorofluorescein diacetate (DCFDA, Invitrogen Inc., Carlsbad, CA, USA). DCFDA is a molecular probe offering derivatives of reduced fluorescein as cell-permeant indicators for ROS. Briefly, cells were seeded in 96-well plates (8000 cells per well) to obtain 70%~80% confluence on the day of the experiment. After incubation overnight, ROS were detected by adding 5 µM DCFDA for 1 h in darkness. The plates were placed in a multimode microplate reader (TECAN Group Ltd., Männedorf, Switzerland) to measure the emitted fluorescence at Ex/Em = 495/529 nm.

### 2.6. Radiation Source

Cells were irradiated using a cabinet RS 2000 Biological Irradiator (Rad Source Technologies, Inc., Suwanee, GA, USA). The radiation dose rate was 290 cGy/min.

### 2.7. Colony Formation Assay

Cells were trypsinized and resuspended in T-25 flasks for radiation exposure at different doses. The T-25 flasks were placed on ice immediately after radiation exposure. Cells were seeded into 6 cm dishes in technical triplicates and maintained in an incubator without disturbance. After 14 days of incubation, the 6 cm dishes were collected, gently washed with 1× phosphate-buffered saline (PBS), and stained with 0.02% crystal violet for 10 min. The dishes were subjected to microscopic examination for quantification of colony numbers (each colony contained more than 50 cells). The plating efficiency was determined as the ratio of the number of colonies divided by the number of seeded cells. The surviving fraction was determined from the ratio of plating efficiency of the irradiated cells compared to the unirradiated controls.

### 2.8. Patients and Tissue Samples

Nineteen cases of human head and neck squamous cell carcinoma and corresponding noncancerous tissues used in this study were obtained from patients who underwent surgical resection (Supplementary Table S1). These samples were immediately snap frozen in liquid nitrogen and stored at −80 °C until RNA extraction. The use of human tissue samples was approved by the Institutional Review Board of Taipei Veteran General Hospital, Taiwan (No. 2018-06-001BC).

### 2.9. Western Blot Analysis

Protein lysates were prepared in EBC lysis buffer containing 1% protease inhibitor (Sigma-Aldrich Co., St. Louis, MO, USA). Equal amounts of total protein were subjected to sodium dodecyl sulfate-polyacrylamide gel electrophoresis (SDS-PAGE) and then electrotransferred onto nitrocellulose membranes (Bio-Rad Laboratories Inc., Hercules, CA, USA). The blots were incubated with blocking reagent (TBS-T with 4% skim milk) at room temperature for 1 h and then hybridized with primary antibodies overnight at 4 °C. Signals were illuminated using Enhanced Chemiluminescence reagent (Bio-Rad Laboratories Inc., Hercules, CA, USA) and recorded on an Image-Quant LAS-4000 imaging system (GE Healthcare, Chicago, IL, USA). The antibodies used in this study included anti-SESN2 antibody (GeneTex Inc., Alton Pkwy Irvine, CA, USA; dilution = 1:1000), anti-heme oxygenase 1 (HO-1) antibody (Santa Cruz Biotechnology Inc., Dallas, TX, USA; dilution = 1:100), and anti-GAPDH (Thermo Fisher Scientific, Waltham, MA, USA; dilution = 1:5000).

### 2.10. Luciferase Activity Assay

Cells were seeded in 6-well plates for 24 h. The miR-182-5p mirVana mimics (Thermo Fisher Scientific Inc., Waltham, MA, USA), pGL4.10-Luc-SESN2 3’UTR-wild type vector and 3’UTR-mutant vector (plasmid construction was performed based on our previous report) [43] were separately cotransfected into cells using INTERFERin^®^ siRNA/miRNA transfection reagent (Polyplus, Huntingdon, UK) according to the manufacturer’s instructions. Luciferase activities were measured using a multimode microplate reader (TECAN Group Ltd., Männedorf, Switzerland), and signals were normalized to the protein concentration.

### 2.11. Cell Viability Assay

Cells were seeded in 96-well plates after transfection with SESN2 siRNA (Cat. #AM16708, Thermo Fisher Scientific Inc., Waltham, MA, USA) for 48 h. After removal of the supernatant, 5 mg/mL 3-(4,5-dimethylthiazol-2-yl)-2, 5-diphenyltetrazolium bromide (MTT) solution (Sigma-Aldrich, St. Louis, MO, USA) was mixed with serum-free medium and incubated for 2~4 h. The produced crystals were dissolved in dimethyl sulfoxide (DMSO). The plate was measured at 570 nm using an ELISA reader (Bio-Tek Instrument, Winooski, VT, USA).

### 2.12. Xenograft Tumor Model

The animal experiment was conducted by following the guidelines of the Institutional Animal Care and Utilization Committee (IACUC number: 1070917). Male BALB/c nude mice aged 6 weeks were purchased from the National Laboratory Animal Center (NLAC, Taipei, Taiwan). For the tumor growth assay, 1 × 10^6^ cells were resuspended in OPTI-MEM (Sigma-Aldrich, St. Louis, MO, USA) and injected into the right thighs of mice (*n* = 5). The tumor volume was monitored using caliper and calculated using the formula (width^2^ × length)/2.

### 2.13. Statistical Analysis

Statistical analysis was performed using GraphPad Prism 6.0 (GraphPad Software, San Diego, CA, USA). All data are presented as the mean ± standard deviation (SD) of independent experiments. Student’s t-test was used for statistical analysis. Two-way analysis of variance (ANOVA) was used to compare the growth curves of xenograft tumors. The survival rates were determined by the Kaplan–Meier method with the log-rank test. A *p*-value < 0.05 was considered to be significant.

## 3. Results

### 3.1. In Silico Data Prediction of the Expression of the miR-182/96/183 Cluster in HNSCC Patients

**Figure 1:**
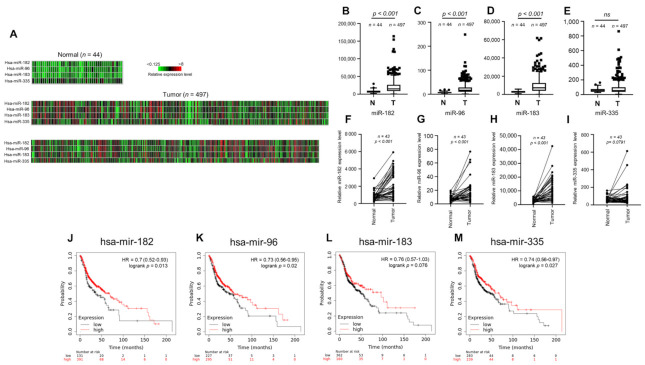
Expression of the miR-182/96/183 cluster in HNSCC patients and survival analysis of patients stratified by miR-182/96/183 cluster expression. (**A**) Heatmaps of miR-182, miR-96, miR-183 and miR-335 expression in normal tissues and HNSCC tissues in the TCGA microRNA sequencing database (TCGA-HNSC). (**B**–**E**) Comparison of miR-182, miR-96, miR-183 and miR-335 expression in the TCGA-HNSC tumor and normal tissues. (**F**–**I**) The expression of miR-182, miR-96, miR-183, and miR-335 in 43 tumor tissues paired with corresponding adjacent normal tissues in the TCGA-HNSC dataset. (**J**–**M**) Comparison of overall survival in patients with high or low expressions of miR-182, miR-96, miR-183 and miR-335.

To investigate the clinical relevance of miR-182/96/183 cluster expression in HNSCC, we first examined the miRNA expression profile using the public TCGA database. A heatmap was used to compare the expression of the miR-182/96/183 cluster and colocalized miR-335 in HNSCC cancer normal controls (Figure 1A). The results of heatmap quantification showed that each member of the miR-182/96/183 cluster was upregulated in HNSCC tissues (Figure 1B–D), but miR-335 was not (Figure 1E). The expression levels of these microRNAs in HNSCC tissues were also compared with those in corresponding adjacent normal tissues, and the results also showed higher expression of the miR-182/96/183 cluster in HNSCC tissues than in surrounding tissues (Figure 1F–H). This difference was also not significant for miR-335 (Figure 1I). We further investigated whether high expression of these microRNAs influenced the survival rate of HNSCC patients, as the miR-182/96/183 cluster was regarded as an oncogenic cluster in most cancers [49]. Surprisingly, the Kaplan–Meier method with the log-rank test showed that high expression levels of miR-182, miR-96 and miR-335 were individually associated with better overall survival rates in HNSCC (Figure 1J–M). Therefore, current data suggest that the miR-182/96/183 cluster is also highly expressed in HNSCC, but its role in tumor biology needs to be further examined.

### 3.2. Radiotherapy Upregulates miR-182 to Induce Oxidative Stress and Enhances Radiosensitivity in HNSCC Cells

**Figure 2:**
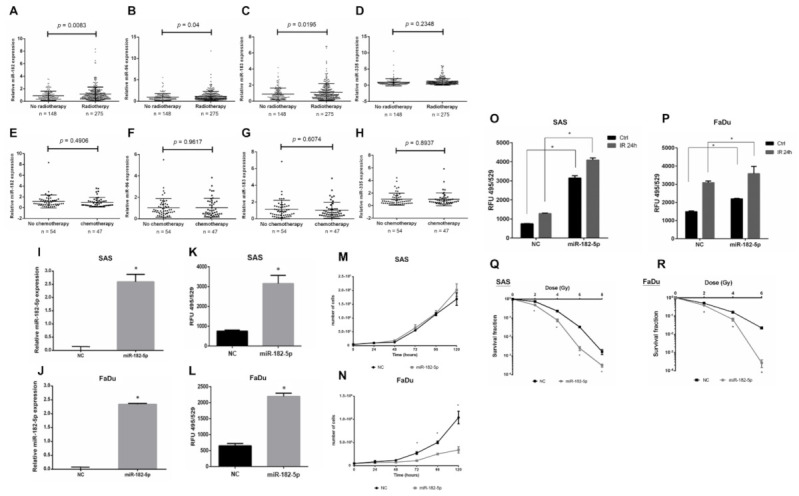
Effects of high miR-182 expression on the radiosensitivity of HNSCC cells. (**A**–**D**) Expression of miR-182, miR-96, miR-183 and miR-335 in HNSCC patients with or without radiotherapy using the TCGA-HNSC dataset. (**E**–**H**) Expression of miR-182, miR-96, miR-183 and miR-335 in HNSCC patients with or without chemotherapy using the TCGA-HNSC dataset. (**I**,**J**) qPCR analysis of miR-182-5p expression in SAS cells and FaDu cells transduced with the pGIPZ-lentiviral shRNAmir constructs, respectively. (**K**,**L**) Quantification of the ROS levels in cells with or without overexpression of miR-182-5p using the DCFDA method. (**M**,**N**) Comparison of cell growth rates between cells with and without overexpression of miR-182-5p. (**O**,**P**) Comparison of radiation effects (IR: 8 Gy) on the intracellular ROS level in cells with or without overexpression of miR-182-5p. (**Q**,**R**) Analysis of surviving fractions of SAS cells and FaDu cells exposed to different doses of X-rays with or without miR-182-5p overexpression, respectively. *: *p* < 0.05.

Radiotherapy is a major treatment for HNSCC, but the role of the miR-182/96/183 cluster in the response to radiation has not been well elucidated. We analyzed the expression of this cluster in HNSCC patients with or without radiotherapy using the TCGA-HNSC dataset. The results showed an upregulation of the miR-182/96/183 cluster in patients who received radiotherapy compared to the control, and miR-182 expression showed the lowest *p* value by *t* test (Figure 2A–C). On the other hand, the miR-335 level did not exhibit a significant change in these two cohorts (Figure 2D). For patients who received chemotherapy, however, the level of neither the miR-182/96/183 cluster nor miR-335 showed a significant change (Figure 2E–H). Because ionizing radiation mainly induces oxidative stress for cell damage, we investigated whether miR-182 would modulate intracellular ROS after irradiation. We first established an miR-182-5p overexpression model by stably transducing the pGIPZ lentiviral shRNAmir constructs into SAS cells and FaDu cells. The expression of exogenous miR-182-5p in both cell types was confirmed using qPCR (Figure 2I,J). The levels of ROS were significantly increased by overexpression of miR-182-5p using the DCFDA quantification method (Figure 2K,L). However, miR-182-5p overexpression reduced the growth rate of FaDu cells more robustly than that of SAS cells (Figure 2M,N). Additionally, overexpression of miR-182-5p significantly increased the radiation-induced ROS level in both HNSCC cell types (Figure 2O,P). Finally, overexpression of miR-182-5p was demonstrated to enhance the radiosensitivity of both SAS cells and FaDu cells by reducing the survival fractions using the colony formation assay (Figure 2Q,R). These data suggest that miR-182 may modulate radiation-induced oxidative stress to influence the radiosensitivity of HNSCC cells.

### 3.3. Characterization of miR-182-5p Expression and Radiation Response in HNSCC Cell Lines

**Figure 3:**
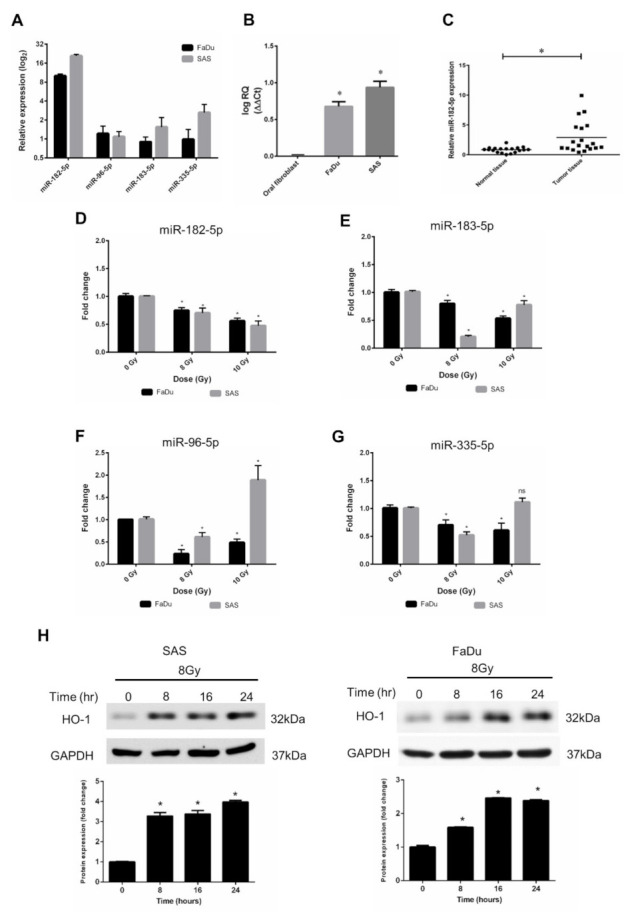
Effects of radiation on the expression of the miR-182/96/183 cluster in HNSCC cells. (**A**) Comparison of endogenous miR-182, miR-96, miR-183, and miR-335 expression in FaDu cells and SAS cells using qPCR analysis. (**B**) Comparison of the endogenous expression of miR-182-5p in normal oral fibroblasts and HNSCC cells. (**C**) Use of surgically excised clinical samples to compare the expression of miR-182-5p between HNSCC tissues and adjacent non-neoplastic tissues (*n* = 19). (**D**–**G**) Effects of X-rays on the expression of miR-182-5p, miR-96-5p, miR-183-5p and miR-335-5p in HNSCC cells. *: *p* < 0.05; ns: non-significant. (**H**) Western blot analysis of HO-1 expression in SAS cells and FaDu cells after 8 Gy X irradiation for up to 24 h. The levels of HO-1 were normalized to GAPDH.

According to the results of bioinformatic analysis using the TCGA database, we validated the expression levels of miR-96, miR-182, miR-183, and miR-335 in human HNSCC cell lines. FaDu cells (hypopharyngeal cancer cells) and SAS cells (tongue cancer cells) were used, and the data showed that miRNA-182-5p expression levels in both cell types were higher than those of other microRNAs (Figure 3A). Consistent with the bioinformatic analysis results, the miR-182-5p level in HNSCC cells was higher than that in normal oral fibroblasts (Figure 3B). In addition, we collected surgically excised clinical HNSCC tissues with adjacent normal tissues and found that miR-182-5p was also upregulated in tumor tissues compared to normal tissues (Figure 3C). The effects of radiation on the expression of the miR-182/96/183 cluster in both cell lines showed that miR-182-5p was downregulated with escalated doses in both cell lines (Figure 3D). The other microRNAs did not show similar patterns after ionizing irradiation (Figure 3E–G). Heme oxygenase-1 (HO-1), an oxidative stress-induced molecule with antioxidant function [50], was induced by radiation in both cell lines (Figure 3H). As overexpression of miR-182-5p increased the ROS level and enhanced radiosensitivity in HNSCC cells, these findings imply that miR-182-5p might regulate antioxidant mechanisms to modulate the radiation response in cells.

### 3.4. MiR-182-5p Targets the Antioxidant Molecule SESN2 in Response to Ionizing Radiation

**Figure 4:**
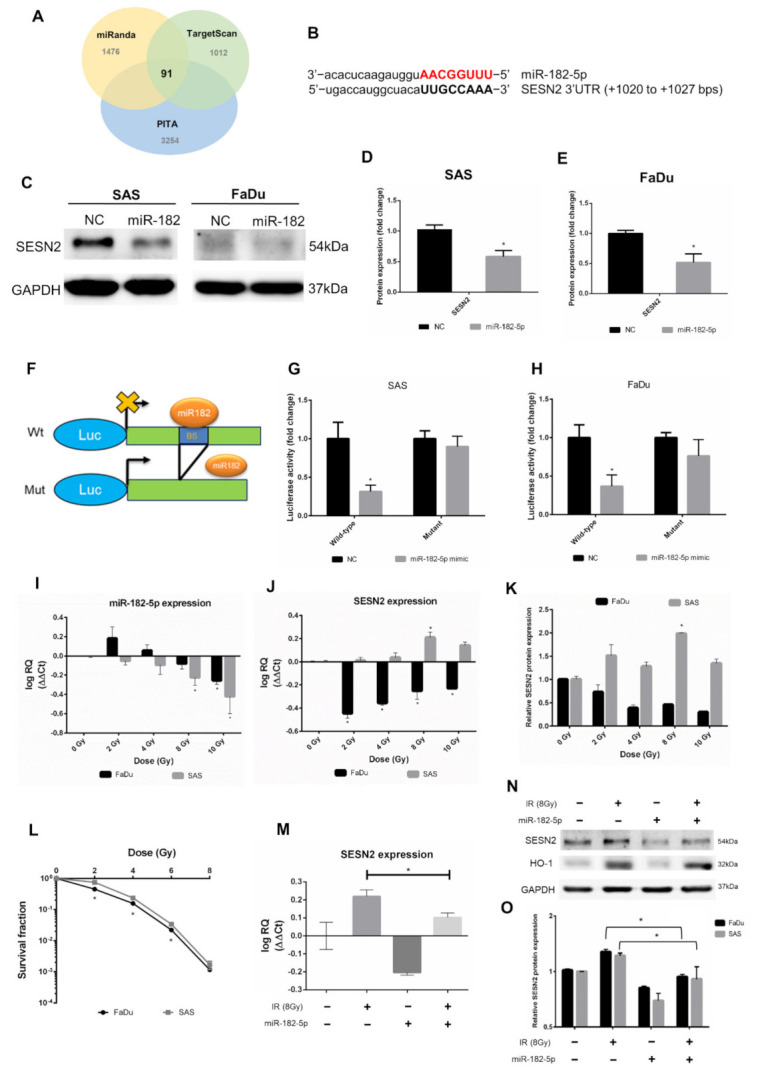
Targeting the antioxidant SESN2 gene by miR-182-5p dose-dependent irradiation. (**A**) Core analysis was performed using the Venn diagram. Three microRNA databases were analyzed to predict potent mRNA interactions with miR-182-5p (CLIP-seq Number >10). (**B**) Sequence complementary between miR-182-5p and the 3’UTR of SESN2. (**C**) Western blot analysis for detection of the expression of SESN2 in HNSCC cells with or without transduction of miR-182-5p. (**D**,**E**) Densitometric quantification of blots, and the levels of SESN2 were normalized to that of GAPDH in both cell lines. The blots were triplicates. (**F**) Illustration of wild-type and mutant 3’UTR of SESN2 mRNA fused to luciferase reporter gene assay. BS: miR-182 binding sites. (**G**,**H**) Quantification of luciferase activity by measuring the photon flux of SAS cells and FaDu cells cotransfected with miR-182-5p mimic and wild-type or mutant reporter constructs, respectively. (**I**) qPCR analysis of miR-182-5p expression in SAS cells and FaDu cells exposed to different doses of radiation. (**J**) The expression of SESN2 mRNA in SAS cells and FaDu cells exposed to different doses of radiation. (**K**) Quantification of blots of SESN2 protein normalized to GAPDH in SAS cells and FaDu cells exposed to different doses of radiation. (**L**) Comparison of the cell survival fractions between irradiated SAS cells and FaDu cells using the colony formation assay. (**M**) Effects of miR-182-5p mimic on the expression of SESN2 mRNA with or without a combination of radiation in SAS cells. (**N**) Effects of miR-182-5p mimic on the expression of SESN2 and HO-1 protein with or without a combination of radiation. (**O**) Densitometric quantification of SESN2 protein expression following miR-182-5p overexpression with or without a combination of radiation. The levels of SESN2 were normalized to that of GAPDH. *: *p* < 0.05.

We next investigated potent downstream targets of miR-182-5p. The predicted mRNAs targeted by miR-182-5p were collected from three microRNA target prediction tools, including miRanda, TargetScan and PITA (cutoff value set as CLIP ≥ 10). The Venn diagram showed that 91 mRNAs were copredicted as targets of miR-182-5p (Figure 4A). The numbers of copredicted targets of miR-96-5p and miR-183-5p were 113 and 64, respectively (Supplementary Figure S1). We found that in 91 miR-182-5p-targeted mRNAs, SESN2 was known to be resistant to oxidative stress by cellular oxidant detoxification according to the DAVID database (Supplementary Table S2). However, SESN2 was not among the copredicted targets of miR-183-5p and miR-96-5p. The sequence of miR-182-5p complementary to the binding site of the SESN2 3’UTR was identified (Figure 4B). Overexpression of miR-182-5p in SAS cells and FaDu cells resulted in a reduction in the SESN2 protein level, even though the basal level of SESN2 in FaDu cells was not high (Figure 4C). These results were then quantified by densitometry (Figure 4D,E). A reporter assay was performed using a luciferase reporter gene fused to the wild-type or mutant 3’UTR of SESN2 mRNA with or without the miR-182-5p binding site (Figure 4F). The results showed that the luciferase activity was inhibited in cells transfected with reporter constructs containing the wild-type 3’UTR of SESN2, but not in cells transfected with reporter constructs containing the mutant 3’UTR (Figure 4G,H). We next sought to determine whether radiation would influence the interaction between miR-182-5p and SESN2 by analyzing RNA expression levels. The qPCR results showed that miR-182-5p expression was induced by low-dose radiation but was suppressed by increased doses in FaDu cells, while dose-dependent downregulation of miR-182-5p was observed in SAS cells (Figure 4I). The expression of SESN2 mRNA was dramatically downregulated in FaDu cells at low doses but was gradually recovered by increased doses, while SESN2 mRNA was induced in a dose-dependent manner up to 8 Gy (Figure 4J). Western blot analysis also demonstrated that the SESN2 protein was reduced and induced in FaDu cells and SAS cells exposed to radiation, respectively (Supplementary Figure S2 and Figure 4K). These results suggest that radiation regulates miR-182-5p-mediated SESN2 expression at the posttranscriptional level. Interestingly, we also found that the intrinsic radiosensitivity of FaDu cells was higher than that of SAS cells (Figure 4L). Additionally, we showed that radiation-induced SESN2 transcription was repressed by transfection of the miR-182-5p mimic in SAS cells (Figure 4M). The change in the SESN2 transcript level was also reflected in the SESN2 protein levels, but HO-1 was not influenced (Figure 4N). MiR-182-5p-mediated suppression of radiation-induced SESN2 was also detected in FaDu cells (Figure 4O). Together, these data suggest that miR-182-5p regulates radiation-induced antioxidant effects by targeting the SESN2 transcript. 

### 3.5. Effects of SESN2 Knockdown on the ROS Level and the Viability of HNSCC Cells Expressing Different Basal Levels of SESN2

**Figure 5:**
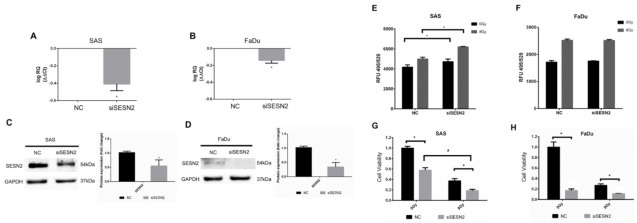
Effects of SESN2 knockdown on ROS levels and HNSCC cell viability. (**A**,**B**) Quantification of SESN2 mRNA in SAS cells and FaDu cells transduced with SESN2 siRNA (siSESN2) by qPCR. (**C**,**D**) Detection of SESN2 protein expression in SAS cells and FaDu cells after transduction of siSESN2, respectively. The levels of SESN2 were normalized to GAPDH. *: *p* < 0.05. (**E**,**F**) Measurement of ROS levels in HNSCC cells with or without transduction of siSESN2. (**G**,**H**) Comparison of cell viability in SAS cells and FaDu cells with or without siSESN2 transduction using an MTT assay. *, #: *p* < 0.05.

We next silenced the expression of SESN2 in SAS cells and FaDu cells to validate the role of SESN2 in the modulation of oxidative stress. The SESN2 mRNAs of SAS cells and FaDu cells were silenced by siRNA and quantified by qPCR (Figure 5A,B). The protein levels of SESN2 were further confirmed by Western blot analysis (Figure 5C,D). Notably, the basal level of SESN2 in SAS cells was higher than that in FaDu cells. The ROS level was then compared in cells with or without knockdown of SESN2, and the results showed that the ROS level increased in SAS cells transfected with SESN2 siRNA (Figure 5E). Knockdown of SESN2 also increased radiation-induced ROS in SAS cells. However, knockdown of SESN2 did not increase the ROS level in FaDu cells with or without exposure to ionizing irradiation (Figure 5F). In addition, knockdown of SESN2 enhanced the radiation-induced suppression of SAS cell viability (Figure 5G). Although knockdown of SESN2 also significantly reduced the viability of FaDu cells, it did not enhance the cytotoxic effects of radiation (Figure 5H). It is speculated that such a small response in ROS level and radiation effects in FaDu cells might be due to the low expression of SESN2. 

### 3.6. Overexpression of miR-182-5p Suppresses Tumorigenesis of HNSCC Cells

**Figure 6:**
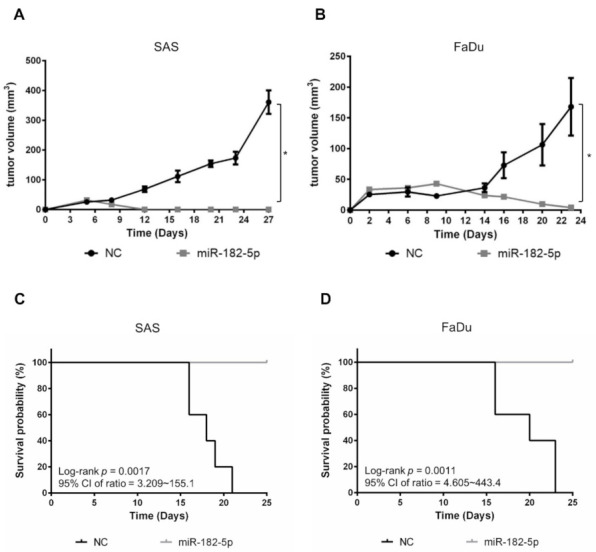
Effects of miR-182-5p overexpression on the tumorigenesis of HNSCC cells in vivo. Tumor sizes were measured at different time points with a caliper in nude mice implanted with (**A**) SAS cells or (**B**) FaDu cells in the right thigh (*n* = 5). MiR-182-5p was transduced into cells using lentiviral transduction as described in the Materials and Methods section. (**C**,**D**) The Kaplan–Meier method with the log-rank test was used to measure the survival of xenograft tumors with or without stably transduced miR-182-5p. *: *p* < 0.05.

We next examined whether miR-182-5p would influence the growth of HNSCC cell-formed xenograft tumors. SAS cells and FaDu cells with or without stably transduced miR-182-5p were separately implanted in nude mice subcutaneously. The results showed that tumor growth was dramatically suppressed by overexpression of miR-182-5p (Figure 6A,B). Moreover, the survival rates were also improved by overexpression of miR-182-5p in both HNSCC cell types (Figure 6C,D). Thus, the current data indicate that high expression of miR-182 is unfavorable for tumorigenesis of HNSCC cells.

### 3.7. Clinical Significance of the miR-182-5p and SESN2 Regulatory Axes in Radiotherapy

**Figure 7:**
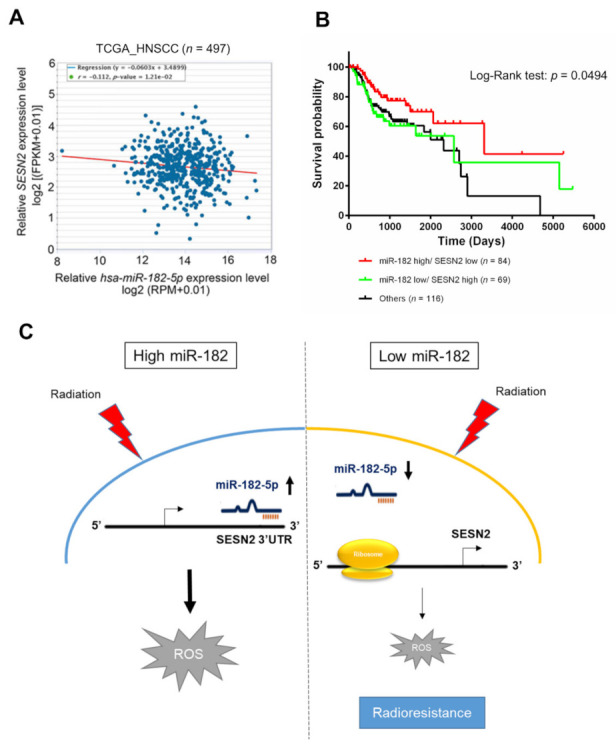
Correlative analysis of miR-182-5p and SESN2 expression on the survival rates of HNSCC patients who received radiotherapy. (**A**) Scatter plot showing a trend of negative correlation between hsa-miR-182-5p and SESN2 mRNA using RNA-Seq analysis in the TCGA-HNSC dataset. (**B**) Comparison of overall survival rates in HNSCC patients who received radiotherapy. The expression of hsa-miR-182 combined with SESN2 gene expression was stratified by three groups: hsa-miR182 high with SESN2 low, hsa-miR-182 low plus SESN2 high, and others. (**C**) Schematic model showing the mechanism by which the miR-182-5p/SESN2 regulatory pathway mediates radiation-induced antioxidant effects and the radiation response.

Whether miR-182-5p targeting of the SESN2 gene is clinically relevant in HNSCC patients was then assessed. TCGA database analysis revealed a trend toward a negative correlation between miR-182-5p expression and SESN2 expression in HNSCC patients that was verified using Spearman correlation analysis (r = −0.112, *p* = 0.0121) (Figure 7A). Furthermore, we integrated the levels of the hsa-miR-182 and SESN2 transcripts and determined their association with the survival rate of HNSCC patients treated with radiotherapy. High expression of miR-182 combined with low expression of SESN2 correlated with a better overall survival rate than reverse expression patterns or others in radiotherapy-treated HNSCC patients (Figure 7B). However, neither miR-182 nor SESN2 significantly influenced the survival rates of HNSCC patients receiving radiotherapy (Supplementary Figure S3). Considering these results collectively, we propose a tentative model in which highly expressed miR-182-5p increases oxidative stress and enhances radiosensitivity by suppressing antioxidant effects via regulation of SESN2 expression (Figure 7C).

## 4. Discussion

Radiotherapy is a double-edged sword that is mainly designed for killing tumors but also induces unexpected adverse effects, including acquired radioresistance. In HNSCC, accumulated literature has demonstrated that radioresistance is associated with recurrent malignancy, which is also linked to high cell motility and invasiveness [6,51,52,53]. This property is correlated with cancer stem cells (CSCs), a rare population (1–5%) of HNSCC cells implicated as being associated with radioresistance, recurrence and metastasis [54]. Additionally, the etiology of radioresistance in HNSCC includes activation of DNA damage repair pathways [55,56], inactivation of p53 [7,57], and overexpression of the epidermal growth factor receptor (EGFR) [58,59]. In this study, we found that radiation could repress miR-182-5p and lead to an antioxidant effect via up-regulation of SESN2 in HNSCC cells. Ionizing radiation has been reported to induce SESN2 expression in human glioblastoma U87 cells [60]. Knockdown of SESN2 not only increases oxidative stress but also sensitizes U87 cells to ionizing radiation [60]. Furthermore, we have previously found that arsenic trioxide (ATO) can concomitantly induce oxidative stress and SESN2 expression through the suppression of miR-182-5p [43]. Here we further showed that over-expression of miR-182-5p inhibited SESN2 expression was associated with the enhancement of radiosensitivity. A radiation induced antioxidant SESN2 protein could be significantly suppressed by transduced miR-182-5p. We also found that high endogenous SESN2 expression was more responsive to knockdown of SESN2 enhanced oxidative stress and cell death induced by radiation. These results suggest that suppression of antioxidant pathway by miR-182-5p may be important for mediating the efficacy of chemo-radiotherapy.

Similar to previous reports [49,61], TCGA analysis also showed that the miR-182/96/183 cluster was upregulated in HNSCC patients. Paradoxically, high expression of this microRNA cluster is associated with better survival rates. According to this phenomenon, it seems that the radiotherapy-induced miR-182/96/183 cluster in HNSCC could be a good prognostic factor. By focusing on miR-182-5p, current results partially agree with some reports defining the role of miR-182-5p as a tumor suppressor rather than an oncogene in HNSCC [62,63]. On the other hand, a hypothesis of “good oncogenes” has been proposed, including FGF18, WIN1, KIT, and STAT1 [64]. Oncogene-induced apoptosis has also been reported [65]. As miR-182-5p is highly expressed in HNSCC, the possibility that miR-182-5p remains an oncomiR cannot be excluded. Although radiation suppressed the expression of miR-182-5p, enforced expression of miR-182-5p further enhanced radiosensitivity but not radioresistance. Our data also showed that overexpression of miR-182-5p was sufficient to inhibit the tumorigenesis of HNSCC cells, regardless of its basal level in a certain cell type. In summary, the intrinsic function of miR-182-5p in HNSCC is still obscure, but overexpression of miR-182-5p should be an adverse effect on cell viability.

MiR-182-5p has been reported to prevent oxidative stress-induced damage in immune cells and lens epithelial cells [26,27]. Additionally, miR-182-5p can attenuate oxidative stress-mediated nonalcoholic steatohepatitis in vivo [66]. Considering these results with ours, the effect of miR-182-5p on oxidative stress may be opposite in cancerous cells and normal cells. This implies that a combination of miR-182-5p and radiotherapy is beneficial for tumor control, as it would increase the cytotoxic effects on tumor cells but protect normal cells against oxidative stress. More evidence is required to support this hypothesis.

MicroRNAs commonly target a variety of mRNAs via complementary 3’-UTRs. Different target-prediction tools use specific algorithms to predict and rank the interactive power of microRNAs and mRNAs. Here, we used three different online prediction tools and showed that 91 genes were predicted by all three tools. SESN2 was one identified gene that can exhibit antioxidant properties. We also found that SESN3 was predicted as a target in miRDB, but it was not included in the other databases. Krishnan et al. demonstrated that miR-182-5p can target a gene network for homologous recombination DNA repair (HRR) [25]. This finding implies that miR-182-5p attenuates DNA repair capacity and sensitizes cells to DNA damage. Radiation is known to induce DNA damage followed by HRR and nonhomologous end joining (NHEJ) repair. It is speculated that both antioxidant effects and altered DNA repair capacity mediated by miR-182-5p may influence radiosensitivity in cancer cells.

Here, we used SAS cells and FaDu cells to compare the effects of miR-182-5p overexpression on radiosensitivity and ROS levels. Although the bioinformatic analysis showed that radiotherapy induced the expression of miR-182, both cell lines showed a reduction in miR-182-5p after ionizing irradiation. Additionally, the basal levels of miR-182-5p and SESN2 were higher in SAS cells than in FaDu cells, suggesting that endogenous SESN2 will also be regulated by other mechanisms in different HNSCC cell types. Although the p53 tumor suppressor is known to transactivate the expression of SESN2, both FaDu cells and SAS cells harbor the mutant p53 gene [67,68,69]. As overexpression of miR-182-5p enhanced radiosensitivity and reduced the SESN2 level in both cell lines, it may exert its potent therapeutic effects via p53-independent mechanisms. Further investigation is essential to confirm this phenomenon.

The reciprocal expression of the miR-182-5p and SESN2 genes was demonstrated not only at the cell level but also in a public patient dataset. According to our data, we collected the overall survival of HNSCC patients which received radiotherapy in the TCGA database (Supplementary Figure S3). We found out that the HNSCC patients with a poor prognosis have low expression of miR-182 or high expression of SESN2. However, there are no significant differences in overall survival rates. As we noticed the relationship between miR-182-5p and SESN2, we combined the two situations in overall survival in HNSCC patients that received radiotherapy. We revealed that high expression of miR-182 combined with low expression of SESN2 correlated with a better overall survival rate (Figure 7B). These findings implicated a better therapeutic value in radiotherapy with high ratio of miR-182/SESN2 levels. However, neither molecule influenced the outcome of radiotherapy individually. Because cancer development is complex and associated with multiple genes, a combination of gene signatures has been demonstrated to improve the prognostic value [70,71]. Integration of mRNA and miRNA expression signatures is also not uncommon in cancer prognosis [72]. As miR-182-5p directly recognizes SESN2, the prognostic value of this molecular interaction in radiotherapy may be important for clinical evaluation.

## 5. Conclusions

In summary, radiation induced antioxidant response is associated with the downregulation of miR-182-5p. Overexpression of miR-182-5p enhanced radiation-induced ROS and radiosensitivity. Furthermore, a regulatory pathway linking miR-182-5p and the antioxidant molecule SESN2 was demonstrated by current data. A high expression level of miR-182-5p combined with a low level of SESN2 was also associated with a better survival rate than the inverse expression pattern in HNSCC patients receiving radiotherapy. Taken together, these findings indicate that an enforced expression of miR-182-5p regulates the intracellular redox status and modulates the efficacy of radiotherapy in HNSCC patients. The miR-182-5p/SESN2 axis may be considered a target for the design of radiosensitizers and as a prognostic factor.

## Figures and Tables

**Table 1:** The sequences of primers used for qPCR analysis.

Primers	Sequence
U6_stemloopRT	gTCgTATCCAgTgCAgggTCCgAggTATTCgCACTggATACgACAAAATATggAAC
miR182_stemloopRT	gTCgTATCCAgTgCAgggTCCgAggTATTCgCACTggATACgACAgTgTg
miR96_stemloopRT	gTCgTATCCAgTgCAgggTCCgAggTATTCgCACTggATACgACAgCAAA
miR183_stemloopRT	gTCgTATCCAgTgCAgggTCCgAggTATTCgCACTggATACgACAgTgAA
miR335_stemloopRT	gTCgTATCCAgTgCAgggTCCgAggTATTCgCACTggATACgACACATTT
U6_Forward	TgCgggTgCTCgCTTCggCAgC
miR182_Forward	TgCggTTTggCAATggTAgAAC
miR96_Forward	gCCgCTTTggCACTAgCACATT
miR183_Forward	gCCgCTTATggCACTggTAgAA
miR335_Forward	CACgCgTCAAgAgCAATAACgA
Universal Reverse	CCAgTgCAgggTCCgAggT
SESN2_s	AAgACCCTACTTTCggA
SESN2_as	CTgCCTggAACTTCTCAT
β-actin_Forward	ggAAATCgTgCgTgACATTAAg
β-actin_Reverse	ggCCATCTCTTgCTCgAAgT

## Data Availability

The data presented in this study are available within the article. Other data that support the findings of this study are available upon request from the corresponding author.

## Supplementary Materials

The following are available online at https://www.mdpi.com/article/10.3390/antiox10111808/s1, Figure S1: The Venn diagram involved three databases that predict possible mRNA targeted by miR-96-5p or miR-183-5p (CLIP > 10). Figure S2: Western blot showed SESN2 expression by different dose of X-ray irradiation. Figure S3: Association of miR-182 and SESN2 expression with overall survival in HNSC patients that received radiotherapy. Table S1: The information about 19 cases of HNSCC patients was showed. Table S2: The co-prediction microRNA targets of miR-182-5p involved in miRanda, TargetScan and PITA.
