# Supplementary material for: OncomiR miR-182-5p Enhances Radiosensitivity by Inhibiting the Radiation-Induced Antioxidant Effect through SESN2 in Head and Neck Cancer
Source: Antioxidants (Basel). 2021 Nov 14;10(11):1808. doi: 10.3390/antiox10111808 (PMC8614815; doi:10.3390/antiox10111808)
Supplement: Supplementary file 1 [file antioxidants-10-01808-s001.zip › Supplementary table_v1.pdf]

Supplementary Table S1. The information about 19 cases of HNSCC patients was showed.

**ID**	**Gender**	**Tumor location**	**TNM**
519T	M	Left glottic	pT2N0M0
567T	M	Left tongue	cT4aN2cM0
571T	M	Hypopharyngeal	pT4aN2cM0
573T	M	Left hypopharyngeal	cT2N0M0
574T	M	Right hypopharyngeal	pT3N2bM0
575T	M	Hypopharyngeal	CT2N0M0
576T	M	Lt vallecular	cT3N2cM0
577T	M	Glottis	pT2N0M0
580T	M	Soft palate	T1N2cM0
588T	M	left, Tongue	pT2N1M0
759T	F	Hypopharyngeal	CT2N2BMO
761T	M	Oropharyngeal tonsil	pT2N2bM0
763T	M	RT Retromolar	PT2N0M0
764T	M	Lt tonsil	cT2N0M0
765T	M	Left lower gingiva	pT4aN1M0
767T	M	Right tongue	cT4aN2cM0
768T	M	Right hypopharyngeal	pT4aN2bM0
770T	M	Right supraglottic	pT2N1M
771T	M	Hypopharyngeal	cT4aN3M1

Supplementary Table S2. The co-prediction microRNA targets of miR-182-5p involved in miRanda, TargetScan and PITA.

ID	Gene Name	GOTERM_BP_DIRECT
ARF4	ADP ribosylation factor 4(ARF4)	GO:0006471~protein ADP-ribosylation,GO:0006888~ER to Golgi vesicle-mediated transport,GO:0006890~retrograde vesicle-mediated transport, Golgi to ER,GO:0007173~epidermal growth factor receptor signaling pathway,GO:0007264~small GTPase mediated signal transduction,GO:0007420~brain development,GO:0007612~learning,GO:0015031~protein transport,GO:0016477~cell migration,GO:0031584~activation of phospholipase D activity,GO:0043066~negative regulation of apoptotic process,GO:0043547~positive regulation of GTPase activity,GO:0045176~apical protein localization,GO:0045197~establishment or maintenance of epithelial cell apical/basal polarity,GO:0045944~positive regulation of transcription from RNA polymerase II promoter,GO:0048678~response to axon injury,GO:0060996~dendritic spine development,GO:0061512~protein localization to cilium,GO:2000377~regulation of reactive oxygen species metabolic process,
AMMECR1L	AMMECR1 like(AMMECR1L)	
ACTR2	ARP2 actin related protein 2 homolog(ACTR2)	GO:0006928~movement of cell or subcellular component,GO:0007163~establishment or maintenance of cell polarity,GO:0008356~asymmetric cell division,GO:0016344~meiotic chromosome movement towards spindle pole,GO:0016482~cytosolic transport,GO:0033206~meiotic cytokinesis,GO:0034314~Arp2/3 complex-mediated actin nucleation,GO:0038096~Fc-gamma receptor signaling pathway involved in phagocytosis,GO:0042384~cilium assembly,GO:0048013~ephrin receptor signaling pathway,GO:0051653~spindle localization,
BCL11A	B-cell CLL/lymphoma 11A(BCL11A)	GO:0000122~negative regulation of transcription from RNA polymerase II promoter,GO:0006351~transcription, DNA-templated,GO:0007165~signal transduction,GO:0010976~positive regulation of neuron projection development,GO:0010977~negative regulation of neuron projection development,GO:0016925~protein sumoylation,GO:0030517~negative regulation of axon extension,GO:0032463~negative regulation of protein homooligomerization,GO:0045944~positive regulation of transcription from RNA polymerase II promoter,GO:0048671~negative regulation of collateral sprouting,GO:0048672~positive regulation of collateral sprouting,GO:0050773~regulation of dendrite

		development,GO:2000171~negative regulation of dendrite development,
BAG4	BCL2 associated athanogene 4(BAG4)	GO:0006457~protein folding,GO:0010763~positive regulation of fibroblast migration,GO:0030838~positive regulation of actin filament polymerization,GO:0033138~positive regulation of peptidyl-serine phosphorylation,GO:0033209~tumor necrosis factor-mediated signaling pathway,GO:0043066~negative regulation of apoptotic process,GO:0045785~positive regulation of cell adhesion,GO:0051291~protein heterooligomerization,GO:0051496~positive regulation of stress fiber assembly,GO:0051897~positive regulation of protein kinase B signaling,GO:0071356~cellular response to tumor necrosis factor,GO:0071364~cellular response to epidermal growth factor stimulus,GO:0072659~protein localization to plasma membrane,GO:0090367~negative regulation of mRNA modification,GO:0097178~ruffle assembly,GO:1900034~regulation of cellular response to heat,GO:1903215~negative regulation of protein targeting to mitochondrion,GO:2001145~negative regulation of phosphatidylinositol-3,4,5-trisphosphate 5-phosphatase activity,
BCL2L12	BCL2 like 12(BCL2L12)	GO:0006915~apoptotic process,GO:0045944~positive regulation of transcription from RNA polymerase II promoter,GO:1902166~negative regulation of intrinsic apoptotic signaling pathway in response to DNA damage by p53 class mediator,GO:1990001~inhibition of cysteine-type endopeptidase activity involved in apoptotic process,GO:2000773~negative regulation of cellular senescence,
CEBPA	CCAAT/enhancer binding protein alpha(CEBPA)	GO:0000050~urea cycle,GO:0000122~negative regulation of transcription from RNA polymerase II promoter,GO:0001889~liver development,GO:0001892~embryonic placenta development,GO:0006091~generation of precursor metabolites and energy,GO:0006351~transcription, DNA-templated,GO:0006360~transcription from RNA polymerase I promoter,GO:0006366~transcription from RNA polymerase II promoter,GO:0007005~mitochondrion organization,GO:0007219~Notch signaling pathway,GO:0008203~cholesterol metabolic process,GO:0008285~negative regulation of cell proliferation,GO:0016032~viral process,GO:0019221~cytokine-mediated signaling pathway,GO:0030099~myeloid cell differentiation,GO:0030225~macrophage differentiation,GO:0030324~lung development,GO:0030851~granulocyte differentiation,GO:0032436~positive regulation of proteasomal ubiquitin-dependent protein catabolic process,GO:0042593~glucose homeostasis,GO:0045444~fat cell differentiation,GO:0045600~positive regulation of fat cell

		differentiation,GO:0045669~positive regulation of osteoblast differentiation,GO:0045736~negative regulation of cyclin-dependent protein serine/threonine kinase activity,GO:0045892~negative regulation of transcription, DNA-templated,GO:0045944~positive regulation of transcription from RNA polymerase II promoter,GO:0045945~positive regulation of transcription from RNA polymerase III promoter,GO:0048469~cell maturation,GO:0048839~inner ear development,GO:0050872~white fat cell differentiation,GO:0050873~brown fat cell differentiation,GO:0055088~lipid homeostasis,GO:0071285~cellular response to lithium ion,GO:0071356~cellular response to tumor necrosis factor,GO:0071407~cellular response to organic cyclic compound,
CD164	CD164 molecule(CD164)	GO:0006955~immune response,GO:0007155~cell adhesion,GO:0007157~heterophilic cell-cell adhesion via plasma membrane cell adhesion molecules,GO:0007162~negative regulation of cell adhesion,GO:0007165~signal transduction,GO:0007275~multicellular organism development,GO:0007517~muscle organ development,GO:0008285~negative regulation of cell proliferation,GO:0030097~hemopoiesis,
CD2AP	CD2 associated protein(CD2AP)	GO:0006461~protein complex assembly,GO:0006930~substrate-dependent cell migration, cell extension,GO:0007010~cytoskeleton organization,GO:0007067~mitotic nuclear division,GO:0007165~signal transduction,GO:0016050~vesicle organization,GO:0016337~single organismal cell-cell adhesion,GO:0032911~negative regulation of transforming growth factor beta1 production,GO:0043161~proteasome-mediated ubiquitin-dependent protein catabolic process,GO:0048259~regulation of receptor-mediated endocytosis,GO:0051301~cell division,GO:1900182~positive regulation of protein localization to nucleus,GO:2000249~regulation of actin cytoskeleton reorganization,
CITED2	Cbp/p300 interacting transactivator with Glu/Asp rich carboxy-terminal domain 2(CITED2)	GO:0000122~negative regulation of transcription from RNA polymerase II promoter,GO:0001570~vasculogenesis,GO:0001666~response to hypoxia,GO:0001829~trophectodermal cell differentiation,GO:0001843~neural tube closure,GO:0001889~liver development,GO:0001892~embryonic placenta development,GO:0001947~heart looping,GO:0002089~lens morphogenesis in camera-type eye,GO:0002244~hematopoietic progenitor cell differentiation,GO:0003151~outflow tract morphogenesis,GO:0003156~regulation of organ formation,GO:0003197~endocardial cushion development,GO:0006351~transcription, DNA-templated,GO:0006355~regulation of transcription, DNA-templated,GO:0007179~transforming growth factor beta receptor signaling pathway,GO:0007368~determination of left/right symmetry,GO:0007417~central

		nervous system development,GO:0007422~peripheral nervous system development,GO:0007507~heart development,GO:0007530~sex determination,GO:0007569~cell aging,GO:0008283~cell proliferation,GO:0008584~male gonad development,GO:0009612~response to mechanical stimulus,GO:0010628~positive regulation of gene expression,GO:0010629~negative regulation of gene expression,GO:0021602~cranial nerve morphogenesis,GO:0022409~positive regulation of cell-cell adhesion,GO:0030154~cell differentiation,GO:0030336~negative regulation of cell migration,GO:0030511~positive regulation of transforming growth factor beta receptor signaling pathway,GO:0030851~granulocyte differentiation,GO:0034405~response to fluid shear stress,GO:0035360~positive regulation of peroxisome proliferator activated receptor signaling pathway,GO:0035802~adrenal cortex formation,GO:0035914~skeletal muscle cell differentiation,GO:0043066~negative regulation of apoptotic process,GO:0043627~response to estrogen,GO:0045787~positive regulation of cell cycle,GO:0045892~negative regulation of transcription, DNA-templated,GO:0045893~positive regulation of transcription, DNA-templated,GO:0045944~positive regulation of transcription from RNA polymerase II promoter,GO:0046697~decidualization,GO:0048536~spleen development,GO:0048538~thymus development,GO:0048596~embryonic camera-type eye morphogenesis,GO:0048821~erythrocyte development,GO:0060136~embryonic process involved in female pregnancy,GO:0060349~bone morphogenesis,GO:0060412~ventricular septum morphogenesis,GO:0060971~embryonic heart tube left/right pattern formation,GO:0060972~left/right pattern formation,GO:0061156~pulmonary artery morphogenesis,GO:0061308~cardiac neural crest cell development involved in heart development,GO:0061428~negative regulation of transcription from RNA polymerase II promoter in response to hypoxia,GO:0070986~left/right axis specification,GO:1900164~nodal signaling pathway involved in determination of lateral mesoderm left/right asymmetry,GO:2000020~positive regulation of male gonad development,
DCAF12	DDB1 and CUL4 associated factor 12(DCAF12)	GO:0016567~protein ubiquitination,
FXR1	FMR1 autosomal homolog 1(FXR1)	GO:0006915~apoptotic process,GO:0007517~muscle organ development,GO:0017148~negative regulation of translation,GO:0030154~cell differentiation,GO:2000637~positive regulation of gene silencing by miRNA,

KDELR1	KDEL endoplasmic reticulum protein retention receptor 1(KDELR1)	GO:0006621~protein retention in ER lumen,GO:0006886~intracellular protein transport,GO:0006888~ER to Golgi vesicle-mediated transport,GO:0006890~retrograde vesicle-mediated transport, Golgi to ER,
LSM14A	LSM14A, mRNA processing body assembly factor(LSM14A)	GO:0006417~regulation of translation,GO:0007275~multicellular organism development,GO:0033962~cytoplasmic mRNA processing body assembly,GO:0039529~RIG-I signaling pathway,GO:0060340~positive regulation of type I interferon-mediated signaling pathway,
MET	MET proto-oncogene, receptor tyrosine kinase(MET)	GO:0001886~endothelial cell morphogenesis,GO:0007165~signal transduction,GO:0007166~cell surface receptor signaling pathway,GO:0007169~transmembrane receptor protein tyrosine kinase signaling pathway,GO:0008283~cell proliferation,GO:0010507~negative regulation of autophagy,GO:0018108~peptidyl-tyrosine phosphorylation,GO:0045944~positive regulation of transcription from RNA polymerase II promoter,GO:0048012~hepatocyte growth factor receptor signaling pathway,GO:0048754~branching morphogenesis of an epithelial tube,GO:0050918~positive chemotaxis,GO:0071526~semaphorin-plexin signaling pathway,GO:1901299~negative regulation of hydrogen peroxide-mediated programmed cell death,GO:2001028~positive regulation of endothelial cell chemotaxis,
MYB	MYB proto-oncogene, transcription factor(MYB)	GO:0000122~negative regulation of transcription from RNA polymerase II promoter,GO:0000278~mitotic cell cycle,GO:0006338~chromatin remodeling,GO:0006355~regulation of transcription, DNA-templated,GO:0006366~transcription from RNA polymerase II promoter,GO:0045624~positive regulation of T-helper cell differentiation,GO:0045892~negative regulation of transcription, DNA-templated,GO:0045893~positive regulation of transcription, DNA-templated,GO:0045944~positive regulation of transcription from RNA polymerase II promoter,GO:0051571~positive regulation of histone H3-K4 methylation,GO:0051574~positive regulation of histone H3-K9 methylation,
NCKAP1	NCK associated protein 1(NCKAP1)	GO:0006915~apoptotic process,GO:0007417~central nervous system development,GO:0010592~positive regulation of lamellipodium assembly,GO:0016032~viral process,GO:0016601~Rac protein signal transduction,GO:0038096~Fc-gamma receptor signaling pathway involved in phagocytosis,GO:0048010~vascular endothelial growth factor receptor signaling pathway,GO:2000601~positive regulation of Arp2/3 complex-mediated actin nucleation,
PRDM16	PR/SET domain	GO:0000122~negative regulation of transcription from RNA polymerase II

	16(PRDM16)	promoter,GO:0006351~transcription, DNA-templated,GO:0006355~regulation of transcription, DNA-templated,GO:0022008~neurogenesis,GO:0030512~negative regulation of transforming growth factor beta receptor signaling pathway,GO:0030853~negative regulation of granulocyte differentiation,GO:0034968~histone lysine methylation,GO:0035019~somatic stem cell population maintenance,GO:0043457~regulation of cellular respiration,GO:0043586~tongue development,GO:0045892~negative regulation of transcription, DNA-templated,GO:0045893~positive regulation of transcription, DNA-templated,GO:0050872~white fat cell differentiation,GO:0050873~brown fat cell differentiation,GO:0060021~palate development,GO:0090336~positive regulation of brown fat cell differentiation,
RAB10	RAB10, member RAS oncogene family(RAB10)	GO:0006893~Golgi to plasma membrane transport,GO:0007264~small GTPase mediated signal transduction,GO:0007409~axonogenesis,GO:0016192~vesicle-mediated transport,GO:0016197~endosomal transport,GO:0019882~antigen processing and presentation,GO:0030859~polarized epithelial cell differentiation,GO:0032869~cellular response to insulin stimulus,GO:0043001~Golgi to plasma membrane protein transport,GO:0045200~establishment of neuroblast polarity,GO:0061024~membrane organization,GO:0061467~basolateral protein localization,GO:0071786~endoplasmic reticulum tubular network organization,GO:0072659~protein localization to plasma membrane,GO:0090150~establishment of protein localization to membrane,GO:0097051~establishment of protein localization to endoplasmic reticulum membrane,GO:0098609~cell-cell adhesion,
ARHGDIA	Rho GDP dissociation inhibitor alpha(ARHGDIA)	GO:0006928~movement of cell or subcellular component,GO:0007162~negative regulation of cell adhesion,GO:0007266~Rho protein signal transduction,GO:0008360~regulation of cell shape,GO:0030336~negative regulation of cell migration,GO:0032880~regulation of protein localization,GO:0035023~regulation of Rho protein signal transduction,GO:0043066~negative regulation of apoptotic process,GO:0043547~positive regulation of GTPase activity,GO:0050771~negative regulation of axonogenesis,GO:0050772~positive regulation of axonogenesis,GO:0051056~regulation of small GTPase mediated signal transduction,GO:0071260~cellular response to mechanical stimulus,GO:0071407~cellular response to organic cyclic compound,GO:0071461~cellular response to redox state,GO:0071526~semaphorin-plexin signaling pathway,GO:2000249~regulation of actin cytoskeleton reorganization,

SH3BP5	SH3 domain binding protein 5(SH3BP5)	GO:0006469~negative regulation of protein kinase activity,GO:0007165~signal transduction,GO:0035556~intracellular signal transduction,GO:0061099~negative regulation of protein tyrosine kinase activity,
SMAD7	SMAD family member 7(SMAD7)	GO:0000122~negative regulation of transcription from RNA polymerase II promoter,GO:0001657~ureteric bud development,GO:0006351~transcription, DNA-templated,GO:0006355~regulation of transcription, DNA-templated,GO:0007179~transforming growth factor beta receptor signaling pathway,GO:0010717~regulation of epithelial to mesenchymal transition,GO:0010719~negative regulation of epithelial to mesenchymal transition,GO:0010801~negative regulation of peptidyl-threonine phosphorylation,GO:0010944~negative regulation of transcription by competitive promoter binding,GO:0017015~regulation of transforming growth factor beta receptor signaling pathway,GO:0022409~positive regulation of cell-cell adhesion,GO:0030336~negative regulation of cell migration,GO:0030509~BMP signaling pathway,GO:0030512~negative regulation of transforming growth factor beta receptor signaling pathway,GO:0030514~negative regulation of BMP signaling pathway,GO:0031397~negative regulation of protein ubiquitination,GO:0031398~positive regulation of protein ubiquitination,GO:0032436~positive regulation of proteasomal ubiquitin-dependent protein catabolic process,GO:0032925~regulation of activin receptor signaling pathway,GO:0033137~negative regulation of peptidyl-serine phosphorylation,GO:0034333~adherens junction assembly,GO:0034616~response to laminar fluid shear stress,GO:0034629~cellular protein complex localization,GO:0043433~negative regulation of sequence-specific DNA binding transcription factor activity,GO:0045944~positive regulation of transcription from RNA polymerase II promoter,GO:0048844~artery morphogenesis,GO:0050821~protein stabilization,GO:0051444~negative regulation of ubiquitin-protein transferase activity,GO:0055010~ventricular cardiac muscle tissue morphogenesis,GO:0055117~regulation of cardiac muscle contraction,GO:0060373~regulation of ventricular cardiac muscle cell membrane depolarization,GO:0060389~pathway-restricted SMAD protein phosphorylation,GO:0060394~negative regulation of pathway-restricted SMAD protein phosphorylation,GO:0060412~ventricular septum morphogenesis,GO:0071560~cellular response to transforming growth factor beta stimulus,
TAF15	TATA-box binding protein associated factor 15(TAF15)	GO:0045893~positive regulation of transcription, DNA-templated,

TWISTNB	TWIST neighbor(TWISTNB)	GO:0006361~transcription initiation from RNA polymerase I promoter,GO:0006362~transcription elongation from RNA polymerase I promoter,GO:0006363~termination of RNA polymerase I transcription,GO:0045815~positive regulation of gene expression, epigenetic,
WIPI2	WD repeat domain, phosphoinositide interacting 2(WIPI2)	GO:0000045~autophagosome assembly,GO:0000422~mitophagy,GO:0006497~protein lipidation,GO:0016236~macroautophagy,GO:0034497~protein localization to pre-autophagosomal structure,GO:0044804~nucleophagy,GO:0097352~autophagosome maturation,
XBP1	X-box binding protein 1(XBP1)	GO:0000122~negative regulation of transcription from RNA polymerase II promoter,GO:0001525~angiogenesis,GO:0001889~liver development,GO:0001934~positive regulation of protein phosphorylation,GO:0001935~endothelial cell proliferation,GO:0002639~positive regulation of immunoglobulin production,GO:0006366~transcription from RNA polymerase II promoter,GO:0006511~ubiquitin-dependent protein catabolic process,GO:0006633~fatty acid biosynthetic process,GO:0006914~autophagy,GO:0006955~immune response,GO:0006990~positive regulation of transcription from RNA polymerase II promoter involved in unfolded protein response,GO:0006996~organelle organization,GO:0007517~muscle organ development,GO:0008284~positive regulation of cell proliferation,GO:0010506~regulation of autophagy,GO:0010508~positive regulation of autophagy,GO:0010832~negative regulation of myotube differentiation,GO:0014065~phosphatidylinositol 3-kinase signaling,GO:0015031~protein transport,GO:0016049~cell growth,GO:0030335~positive regulation of cell migration,GO:0030512~negative regulation of transforming growth factor beta receptor signaling pathway,GO:0030968~endoplasmic reticulum unfolded protein response,GO:0031017~exocrine pancreas development,GO:0031062~positive regulation of histone methylation,GO:0031647~regulation of protein stability,GO:0031648~protein destabilization,GO:0031670~cellular response to nutrient,GO:0032008~positive regulation of TOR signaling,GO:0032869~cellular response to insulin stimulus,GO:0034599~cellular response to oxidative stress,GO:0034976~response to endoplasmic reticulum stress,GO:0035356~cellular triglyceride homeostasis,GO:0035470~positive regulation of vascular wound healing,GO:0035924~cellular response to vascular endothelial growth factor stimulus,GO:0036498~IRE1-mediated unfolded protein response,GO:0036500~ATF6-mediated unfolded protein response,GO:0042149~cellular response to glucose

		starvation,GO:0042632~cholesterol homeostasis,GO:0042993~positive regulation of transcription factor import into nucleus,GO:0043066~negative regulation of apoptotic process,GO:0045348~positive regulation of MHC class II biosynthetic process,GO:0045579~positive regulation of B cell differentiation,GO:0045582~positive regulation of T cell differentiation,GO:0045600~positive regulation of fat cell differentiation,GO:0045766~positive regulation of angiogenesis,GO:0045944~positive regulation of transcription from RNA polymerase II promoter,GO:0048010~vascular endothelial growth factor receptor signaling pathway,GO:0048666~neuron development,GO:0051024~positive regulation of immunoglobulin secretion,GO:0051897~positive regulation of protein kinase B signaling,GO:0055089~fatty acid homeostasis,GO:0055092~sterol homeostasis,GO:0060394~negative regulation of pathway-restricted SMAD protein phosphorylation,GO:0060612~adipose tissue development,GO:0060691~epithelial cell maturation involved in salivary gland development,GO:0070059~intrinsic apoptotic signaling pathway in response to endoplasmic reticulum stress,GO:0070373~negative regulation of ERK1 and ERK2 cascade,GO:0071222~cellular response to lipopolysaccharide,GO:0071230~cellular response to amino acid stimulus,GO:0071332~cellular response to fructose stimulus,GO:0071333~cellular response to glucose stimulus,GO:0071353~cellular response to interleukin-4,GO:0071375~cellular response to peptide hormone stimulus,GO:0071498~cellular response to fluid shear stress,GO:0071499~cellular response to laminar fluid shear stress,GO:1900100~positive regulation of plasma cell differentiation,GO:1900102~negative regulation of endoplasmic reticulum unfolded protein response,GO:1900103~positive regulation of endoplasmic reticulum unfolded protein response,GO:1900413~positive regulation of phospholipid biosynthetic process by positive regulation of transcription from RNA polymerase II promoter,GO:1901800~positive regulation of proteasomal protein catabolic process,GO:1901985~positive regulation of protein acetylation,GO:1902236~negative regulation of endoplasmic reticulum stress-induced intrinsic apoptotic signaling pathway,GO:1903071~positive regulation of ER-associated ubiquitin-dependent protein catabolic process,GO:1903489~positive regulation of lactation,GO:1904707~positive regulation of vascular smooth muscle cell proliferation,GO:1904754~positive regulation of vascular associated smooth muscle cell migration,GO:1990418~response to insulin-like growth factor stimulus,GO:1990440~positive regulation of transcription from RNA polymerase

		II promoter in response to endoplasmic reticulum stress,GO:2000347~positive regulation of hepatocyte proliferation,GO:2000353~positive regulation of endothelial cell apoptotic process,GO:2000778~positive regulation of interleukin-6 secretion,
ZFP36L1	ZFP36 ring finger protein like 1(ZFP36L1)	GO:0000165~MAPK cascade,GO:0000288~nuclear-transcribed mRNA catabolic process, deadenylation-dependent decay,GO:0001570~vasculogenesis,GO:0003342~proepicardium development,GO:0006355~regulation of transcription, DNA-templated,GO:0006397~mRNA processing,GO:0006417~regulation of translation,GO:0006915~apoptotic process,GO:0007507~heart development,GO:0008283~cell proliferation,GO:0009611~response to wounding,GO:0010468~regulation of gene expression,GO:0010837~regulation of keratinocyte proliferation,GO:0014065~phosphatidylinositol 3-kinase signaling,GO:0021915~neural tube development,GO:0031086~nuclear-transcribed mRNA catabolic process, deadenylation-independent decay,GO:0031440~regulation of mRNA 3'-end processing,GO:0032869~cellular response to insulin stimulus,GO:0033077~T cell differentiation in thymus,GO:0035264~multicellular organism growth,GO:0038066~p38MAPK cascade,GO:0043488~regulation of mRNA stability,GO:0043491~protein kinase B signaling,GO:0044344~cellular response to fibroblast growth factor stimulus,GO:0045577~regulation of B cell differentiation,GO:0045600~positive regulation of fat cell differentiation,GO:0045616~regulation of keratinocyte differentiation,GO:0045647~negative regulation of erythrocyte differentiation,GO:0045657~positive regulation of monocyte differentiation,GO:0045661~regulation of myoblast differentiation,GO:0048382~mesendoderm development,GO:0051028~mRNA transport,GO:0060710~chorio-allantoic fusion,GO:0060712~spongiotrophoblast layer development,GO:0061158~3'-UTR-mediated mRNA destabilization,GO:0070371~ERK1 and ERK2 cascade,GO:0071320~cellular response to cAMP,GO:0071356~cellular response to tumor necrosis factor,GO:0071364~cellular response to epidermal growth factor stimulus,GO:0071375~cellular response to peptide hormone stimulus,GO:0071385~cellular response to glucocorticoid stimulus,GO:0071456~cellular response to hypoxia,GO:0071472~cellular response to salt stress,GO:0071560~cellular response to transforming growth factor beta stimulus,GO:0072091~regulation of stem cell proliferation,GO:0097403~cellular response to raffinose,GO:1900153~positive regulation of nuclear-transcribed mRNA catabolic process, deadenylation-

		dependent decay,GO:1901991~negative regulation of mitotic cell cycle phase transition,GO:1902172~regulation of keratinocyte apoptotic process,GO:1904582~positive regulation of intracellular mRNA localization,GO:1904628~cellular response to phorbol 13-acetate 12-myristate,
APPL1	adaptor protein, phosphotyrosine interacting with PH domain and leucine zipper 1(APPL1)	GO:0007049~cell cycle,GO:0007165~signal transduction,GO:0008283~cell proliferation,GO:0008286~insulin receptor signaling pathway,GO:0046324~regulation of glucose import,GO:0090003~regulation of establishment of protein localization to plasma membrane,GO:0097192~extrinsic apoptotic signaling pathway in absence of ligand,
ADGRL2	adhesion G protein-coupled receptor L2(ADGRL2)	GO:0007166~cell surface receptor signaling pathway,GO:0007186~G-protein coupled receptor signaling pathway,
AMOTL2	angiomotin like 2(AMOTL2)	GO:0016055~Wnt signaling pathway,GO:0035329~hippo signaling,
ANLN	anillin actin binding protein(ANLN)	GO:0000281~mitotic cytokinesis,GO:0000921~septin ring assembly,GO:0002244~hematopoietic progenitor cell differentiation,GO:0007067~mitotic nuclear division,GO:0007096~regulation of exit from mitosis,GO:0090521~glomerular visceral epithelial cell migration,GO:0098609~cell-cell adhesion,
ANKRD27	ankyrin repeat domain 27(ANKRD27)	GO:0015031~protein transport,GO:0035544~negative regulation of SNARE complex assembly,GO:0035646~endosome to melanosome transport,GO:0043547~positive regulation of GTPase activity,GO:0045022~early endosome to late endosome transport,GO:0048812~neuron projection morphogenesis,GO:0050775~positive regulation of dendrite morphogenesis,GO:1990126~retrograde transport, endosome to plasma membrane,
ARRDC3	arrestin domain containing 3(ARRDC3)	GO:0001659~temperature homeostasis,GO:0007165~signal transduction,GO:0031651~negative regulation of heat generation,GO:0043588~skin development,GO:0044252~negative regulation of multicellular organismal metabolic process,GO:0051443~positive regulation of ubiquitin-protein transferase activity,GO:0060613~fat pad development,GO:0071879~positive regulation of adrenergic receptor signaling pathway,GO:0090327~negative regulation of locomotion involved in locomotory behavior,
BCAT2	branched chain amino acid transaminase 2(BCAT2)	GO:0006532~aspartate biosynthetic process,GO:0006550~isoleucine catabolic process,GO:0008152~metabolic process,GO:0008652~cellular amino acid biosynthetic process,GO:0009082~branched-chain amino acid biosynthetic process,GO:0009083~branched-chain amino acid catabolic

		process,GO:0009098~leucine biosynthetic process,GO:0009099~valine biosynthetic process,GO:0010817~regulation of hormone levels,
CAMSAP2	calmodulin regulated spectrin associated protein family member 2(CAMSAP2)	GO:0000226~microtubule cytoskeleton organization,GO:0031175~neuron projection development,GO:0033043~regulation of organelle organization,
CHST10	carbohydrate sulfotransferase 10(CHST10)	GO:0007155~cell adhesion,GO:0016051~carbohydrate biosynthetic process,
CFL1	cofilin 1(CFL1)	GO:0006606~protein import into nucleus,GO:0007010~cytoskeleton organization,GO:0007266~Rho protein signal transduction,GO:0009615~response to virus,GO:0022604~regulation of cell morphogenesis,GO:0030030~cell projection organization,GO:0030036~actin cytoskeleton organization,GO:0030042~actin filament depolymerization,GO:0043066~negative regulation of apoptotic process,GO:0044794~positive regulation by host of viral process,GO:0045792~negative regulation of cell size,GO:0061001~regulation of dendritic spine morphogenesis,
CNNM3	cyclin and CBS domain divalent metal cation transport mediator 3(CNNM3)	GO:0006811~ion transport,
DUSP1	dual specificity phosphatase 1(DUSP1)	GO:0000188~inactivation of MAPK activity,GO:0001706~endoderm formation,GO:0006979~response to oxidative stress,GO:0009416~response to light stimulus,GO:0032355~response to estradiol,GO:0032526~response to retinoic acid,GO:0032870~cellular response to hormone stimulus,GO:0033574~response to testosterone,GO:0035335~peptidyl-tyrosine dephosphorylation,GO:0035556~intracellular signal transduction,GO:0035970~peptidyl-threonine dephosphorylation,GO:0042542~response to hydrogen peroxide,GO:0042981~regulation of apoptotic process,GO:0043065~positive regulation of apoptotic process,GO:0043066~negative regulation of apoptotic process,GO:0043407~negative regulation of MAP kinase activity,GO:0043409~negative regulation of MAPK cascade,GO:0051384~response to glucocorticoid,GO:0051447~negative regulation of meiotic cell cycle,GO:0051591~response to cAMP,GO:0051592~response to calcium ion,GO:0070373~negative regulation of ERK1 and ERK2 cascade,GO:0071850~mitotic cell cycle arrest,GO:0090266~regulation of mitotic cell cycle spindle assembly

		checkpoint,GO:2000279~negative regulation of DNA biosynthetic process,
EVI5	ecotropic viral integration site 5(EVI5)	GO:0006886~intracellular protein transport,GO:0007049~cell cycle,GO:0007275~multicellular organism development,GO:0008283~cell proliferation,GO:0031338~regulation of vesicle fusion,GO:0042147~retrograde transport, endosome to Golgi,GO:0051301~cell division,GO:0090630~activation of GTPase activity,GO:1902017~regulation of cilium assembly,
ELL2	elongation factor for RNA polymerase II 2(ELL2)	GO:0006355~regulation of transcription, DNA-templated,GO:0006368~transcription elongation from RNA polymerase II promoter,GO:0042795~snRNA transcription from RNA polymerase II promoter,
ELL	elongation factor for RNA polymerase II(ELL)	GO:0001701~in utero embryonic development,GO:0006366~transcription from RNA polymerase II promoter,GO:0006368~transcription elongation from RNA polymerase II promoter,GO:0010923~negative regulation of phosphatase activity,GO:0032786~positive regulation of DNA-templated transcription, elongation,GO:0032968~positive regulation of transcription elongation from RNA polymerase II promoter,GO:0042795~snRNA transcription from RNA polymerase II promoter,GO:0042796~snRNA transcription from RNA polymerase III promoter,GO:0045945~positive regulation of transcription from RNA polymerase III promoter,
FAM222B	family with sequence similarity 222 member B(FAM222B)	
FAM49B	family with sequence similarity 49 member B(FAM49B)	
FRS2	fibroblast growth factor receptor substrate 2(FRS2)	GO:0000165~MAPK cascade,GO:0000186~activation of MAPKK activity,GO:0000187~activation of MAPK activity,GO:0001702~gastrulation with mouth forming second,GO:0001759~organ induction,GO:0003281~ventricular septum development,GO:0007185~transmembrane receptor protein tyrosine phosphatase signaling pathway,GO:0007186~G-protein coupled receptor signaling pathway,GO:0007405~neuroblast proliferation,GO:0008543~fibroblast growth factor receptor signaling pathway,GO:0008595~anterior/posterior axis specification, embryo,GO:0014066~regulation of phosphatidylinositol 3-kinase signaling,GO:0030900~forebrain development,GO:0036092~phosphatidylinositol-3-phosphate biosynthetic process,GO:0042981~regulation of apoptotic process,GO:0043547~positive regulation of GTPase activity,GO:0046619~optic placode formation involved in camera-type eye formation,GO:0046854~phosphatidylinositol phosphorylation,GO:0048015~phosphatidylinositol-mediated signaling,GO:0050678~regulation of epithelial cell proliferation,GO:0060527~prostate epithelial cord arborization involved in prostate glandular acinus morphogenesis,GO:0070307~lens fiber cell development,GO:0070372~regulation of ERK1 and ERK2 cascade,

FN1	fibronectin 1(FN1)	GO:0001525~angiogenesis,GO:0001932~regulation of protein phosphorylation,GO:0002576~platelet degranulation,GO:0006953~acute-phase response,GO:0007044~cell-substrate junction assembly,GO:0007155~cell adhesion,GO:0007161~calcium-independent cell-matrix adhesion,GO:0008284~positive regulation of cell proliferation,GO:0008360~regulation of cell shape,GO:0009611~response to wounding,GO:0010628~positive regulation of gene expression,GO:0010952~positive regulation of peptidase activity,GO:0018149~peptide cross-linking,GO:0022617~extracellular matrix disassembly,GO:0030198~extracellular matrix organization,GO:0033622~integrin activation,GO:0034446~substrate adhesion-dependent cell spreading,GO:0035987~endodermal cell differentiation,GO:0042060~wound healing,GO:0045773~positive regulation of axon extension,GO:0048146~positive regulation of fibroblast proliferation,GO:0050900~leukocyte migration,GO:0070372~regulation of ERK1 and ERK2 cascade,GO:1904237~positive regulation of substrate-dependent cell migration, cell attachment to substrate,GO:2001202~negative regulation of transforming growth factor-beta secretion,
FNDC3B	fibronectin type III domain containing 3B(FNDC3B)	
FOXF2	forkhead box F2(FOXF2)	GO:0001837~epithelial to mesenchymal transition,GO:0006366~transcription from RNA polymerase II promoter,GO:0030198~extracellular matrix organization,GO:0042249~establishment of planar polarity of embryonic epithelium,GO:0045892~negative regulation of transcription, DNA-templated,GO:0045893~positive regulation of transcription, DNA-templated,GO:0045944~positive regulation of transcription from RNA polymerase II promoter,GO:0048566~embryonic digestive tract development,GO:0048596~embryonic camera-type eye morphogenesis,GO:0048806~genitalia development,GO:0060021~palate development,
FOXN2	forkhead box N2(FOXN2)	GO:0006351~transcription, DNA-templated,GO:0030154~cell differentiation,GO:0035914~skeletal muscle cell differentiation,GO:0045893~positive regulation of transcription, DNA-templated,
GXYLT1	glucoside xylosyltransferase 1(GXYLT1)	GO:0016266~O-glycan processing,GO:0030203~glycosaminoglycan metabolic process,
QSER1	glutamine and serine rich 1(QSER1)	
GRB2	growth factor receptor bound protein 2(GRB2)	GO:0000165~MAPK cascade,GO:0007173~epidermal growth factor receptor signaling pathway,GO:0007265~Ras protein signal

		transduction,GO:0007267~cell-cell signaling,GO:0007411~axon guidance,GO:0007568~aging,GO:0008286~insulin receptor signaling pathway,GO:0008543~fibroblast growth factor receptor signaling pathway,GO:0009967~positive regulation of signal transduction,GO:0014066~regulation of phosphatidylinositol 3-kinase signaling,GO:0016032~viral process,GO:0030154~cell differentiation,GO:0030838~positive regulation of actin filament polymerization,GO:0031295~T cell costimulation,GO:0031623~receptor internalization,GO:0036092~phosphatidylinositol-3-phosphate biosynthetic process,GO:0038095~Fc-epsilon receptor signaling pathway,GO:0038096~Fc-gamma receptor signaling pathway involved in phagocytosis,GO:0038128~ERBB2 signaling pathway,GO:0042059~negative regulation of epidermal growth factor receptor signaling pathway,GO:0042770~signal transduction in response to DNA damage,GO:0043408~regulation of MAPK cascade,GO:0043547~positive regulation of GTPase activity,GO:0046854~phosphatidylinositol phosphorylation,GO:0048015~phosphatidylinositol-mediated signaling,GO:0048646~anatomical structure formation involved in morphogenesis,GO:0050900~leukocyte migration,GO:0051291~protein heterooligomerization,GO:0060670~branching involved in labyrinthine layer morphogenesis,GO:0071479~cellular response to ionizing radiation,GO:2000379~positive regulation of reactive oxygen species metabolic process,
HES1	hes family bHLH transcription factor 1(HES1)	GO:0000122~negative regulation of transcription from RNA polymerase II promoter,GO:0001889~liver development,GO:0003143~embryonic heart tube morphogenesis,GO:0003151~outflow tract morphogenesis,GO:0003266~regulation of secondary heart field cardioblast proliferation,GO:0003281~ventricular septum development,GO:0006351~transcription, DNA-templated,GO:0006355~regulation of transcription, DNA-templated,GO:0006461~protein complex assembly,GO:0007155~cell adhesion,GO:0007219~Notch signaling pathway,GO:0007224~smoothened signaling pathway,GO:0007262~STAT protein import into nucleus,GO:0007399~nervous system development,GO:0008284~positive regulation of cell proliferation,GO:0016477~cell migration,GO:0021537~telencephalon development,GO:0021555~midbrain-hindbrain boundary morphogenesis,GO:0021557~oculomotor nerve development,GO:0021558~trochlear nerve development,GO:0021575~hindbrain

		morphogenesis,GO:0021861~forebrain radial glial cell differentiation,GO:0021984~adenohypophysis development,GO:0030324~lung development,GO:0030513~positive regulation of BMP signaling pathway,GO:0030901~midbrain development,GO:0031016~pancreas development,GO:0035019~somatic stem cell population maintenance,GO:0035910~ascending aorta morphogenesis,GO:0042102~positive regulation of T cell proliferation,GO:0042517~positive regulation of tyrosine phosphorylation of Stat3 protein,GO:0042668~auditory receptor cell fate determination,GO:0043388~positive regulation of DNA binding,GO:0045598~regulation of fat cell differentiation,GO:0045608~negative regulation of auditory receptor cell differentiation,GO:0045747~positive regulation of Notch signaling pathway,GO:0045892~negative regulation of transcription, DNA-templated,GO:0045944~positive regulation of transcription from RNA polymerase II promoter,GO:0045977~positive regulation of mitotic cell cycle, embryonic,GO:0046331~lateral inhibition,GO:0046427~positive regulation of JAK-STAT cascade,GO:0048469~cell maturation,GO:0048538~thymus development,GO:0048667~cell morphogenesis involved in neuron differentiation,GO:0048711~positive regulation of astrocyte differentiation,GO:0048715~negative regulation of oligodendrocyte differentiation,GO:0048844~artery morphogenesis,GO:0050678~regulation of epithelial cell proliferation,GO:0060122~inner ear receptor stereocilium organization,GO:0060164~regulation of timing of neuron differentiation,GO:0060253~negative regulation of glial cell proliferation,GO:0060412~ventricular septum morphogenesis,GO:0060675~ureteric bud morphogenesis,GO:0060716~labyrinthine layer blood vessel development,GO:0061009~common bile duct development,GO:0061106~negative regulation of stomach neuroendocrine cell differentiation,GO:0061309~cardiac neural crest cell development involved in outflow tract morphogenesis,GO:0061626~pharyngeal arch artery morphogenesis,GO:0072012~glomerulus vasculature development,GO:0072049~comma-shaped body morphogenesis,GO:0072050~S-shaped body morphogenesis,GO:0072141~renal interstitial fibroblast development,GO:0072282~metanephric nephron tubule morphogenesis,GO:0090102~cochlea development,GO:0090162~establishment of epithelial cell polarity,GO:0097084~vascular smooth muscle cell development,GO:0097150~neuronal stem cell population maintenance,GO:1903955~positive regulation of protein targeting to

		mitochondrion,GO:2000227~negative regulation of pancreatic A cell differentiation,GO:2000737~negative regulation of stem cell differentiation,GO:2000974~negative regulation of pro-B cell differentiation,GO:2000978~negative regulation of forebrain neuron differentiation,
HOXA9	homeobox A9(HOXA9)	GO:0006351~transcription, DNA-templated,GO:0006355~regulation of transcription, DNA-templated,GO:0007275~multicellular organism development,GO:0007283~spermatogenesis,GO:0007338~single fertilization,GO:0008584~male gonad development,GO:0009952~anterior/posterior pattern specification,GO:0009954~proximal/distal pattern formation,GO:0030879~mammary gland development,GO:0035115~embryonic forelimb morphogenesis,GO:0042118~endothelial cell activation,GO:0045638~negative regulation of myeloid cell differentiation,GO:0048706~embryonic skeletal system development,GO:0060065~uterus development,GO:0060216~definitive hemopoiesis,
HSDL1	hydroxysteroid dehydrogenase like 1(HSDL1)	
IVNS1ABP	influenza virus NS1A binding protein(IVNS1ABP)	GO:0006383~transcription from RNA polymerase III promoter,GO:0008380~RNA splicing,GO:0009615~response to virus,GO:2001243~negative regulation of intrinsic apoptotic signaling pathway,
INSIG1	insulin induced gene 1(INSIG1)	GO:0006641~triglyceride metabolic process,GO:0006695~cholesterol biosynthetic process,GO:0006991~response to sterol depletion,GO:0008152~metabolic process,GO:0008203~cholesterol metabolic process,GO:0008283~cell proliferation,GO:0010894~negative regulation of steroid biosynthetic process,GO:0032933~SREBP signaling pathway,GO:0042472~inner ear morphogenesis,GO:0042474~middle ear morphogenesis,GO:0045599~negative regulation of fat cell differentiation,GO:0045717~negative regulation of fatty acid biosynthetic process,GO:0060021~palate development,GO:0060363~cranial suture morphogenesis,GO:1901303~negative regulation of cargo loading into COPII-coated vesicle,
IGF1R	insulin like growth factor 1 receptor(IGF1R)	GO:0006955~immune response,GO:0007165~signal transduction,GO:0007169~transmembrane receptor protein tyrosine kinase signaling pathway,GO:0008284~positive regulation of cell proliferation,GO:0008286~insulin receptor signaling pathway,GO:0014065~phosphatidylinositol 3-kinase signaling,GO:0030335~positive regulation of cell

		migration,GO:0038083~peptidyl-tyrosine autophosphorylation,GO:0043066~negative regulation of apoptotic process,GO:0045740~positive regulation of DNA replication,GO:0046328~regulation of JNK cascade,GO:0046777~protein autophosphorylation,GO:0048009~insulin-like growth factor receptor signaling pathway,GO:0048015~phosphatidylinositol-mediated signaling,GO:0051262~protein tetramerization,GO:0051389~inactivation of MAPKK activity,
KPNB1	karyopherin subunit beta 1(KPNB1)	GO:0000059~protein import into nucleus, docking,GO:0000060~protein import into nucleus, translocation,GO:0006309~apoptotic DNA fragmentation,GO:0006606~protein import into nucleus,GO:0006607~NLS-bearing protein import into nucleus,GO:0006610~ribosomal protein import into nucleus,GO:0006886~intracellular protein transport,GO:0007079~mitotic chromosome movement towards spindle pole,GO:0007080~mitotic metaphase plate congression,GO:0019054~modulation by virus of host process,GO:0030953~astral microtubule organization,GO:0031291~Ran protein signal transduction,GO:0040001~establishment of mitotic spindle localization,GO:0045184~establishment of protein localization,GO:0075733~intracellular transport of virus,GO:0090307~mitotic spindle assembly,
KHDC4	KH Domain Containing 4	
KLHL28	kelch like family member 28(KLHL28)	GO:0042787~protein ubiquitination involved in ubiquitin-dependent protein catabolic process,
MFAP3	microfibrillar associated protein 3(MFAP3)	
NPM1	nucleophosmin(NPM1)	GO:0006281~DNA repair,GO:0006334~nucleosome assembly,GO:0006886~intracellular protein transport,GO:0006913~nucleocytoplasmic transport,GO:0006950~response to stress,GO:0006977~DNA damage response, signal transduction by p53 class mediator resulting in cell cycle arrest,GO:0007098~centrosome cycle,GO:0007165~signal transduction,GO:0007569~cell aging,GO:0008104~protein localization,GO:0008284~positive regulation of cell proliferation,GO:0008285~negative regulation of cell proliferation,GO:0010826~negative regulation of centrosome duplication,GO:0016032~viral process,GO:0032071~regulation of endodeoxyribonuclease activity,GO:0034080~CENP-A containing nucleosome assembly,GO:0042255~ribosome assembly,GO:0043066~negative regulation of apoptotic process,GO:0043085~positive regulation of catalytic activity,GO:0043523~regulation of neuron apoptotic

		process,GO:0044387~negative regulation of protein kinase activity by regulation of protein phosphorylation,GO:0045727~positive regulation of translation,GO:0045740~positive regulation of DNA replication,GO:0045893~positive regulation of transcription, DNA-templated,GO:0046599~regulation of centriole replication,GO:0051092~positive regulation of NF-kappaB transcription factor activity,GO:0051259~protein oligomerization,GO:0051260~protein homooligomerization,GO:0060699~regulation of endoribonuclease activity,GO:0060735~regulation of eIF2 alpha phosphorylation by dsRNA,GO:1902751~positive regulation of cell cycle G2/M phase transition,
NUP50	nucleoporin 50(NUP50)	GO:0006406~mRNA export from nucleus,GO:0006409~tRNA export from nucleus,GO:0006606~protein import into nucleus,GO:0007077~mitotic nuclear envelope disassembly,GO:0010827~regulation of glucose transport,GO:0016032~viral process,GO:0016925~protein sumoylation,GO:0019083~viral transcription,GO:0031047~gene silencing by RNA,GO:0046907~intracellular transport,GO:0075733~intracellular transport of virus,GO:1900034~regulation of cellular response to heat,
PAIP1	poly(A) binding protein interacting protein 1(PAIP1)	GO:0000289~nuclear-transcribed mRNA poly(A) tail shortening,GO:0006413~translational initiation,GO:0045727~positive regulation of translation,GO:0048255~mRNA stabilization,
PAIP2	poly(A) binding protein interacting protein 2(PAIP2)	GO:0007283~spermatogenesis,GO:0007613~memory,GO:0045947~negative regulation of translational initiation,GO:1900271~regulation of long-term synaptic potentiation,
PREPL	prolyl endopeptidase-like(PREPL)	GO:0006508~proteolysis,
PRKCE	protein kinase C epsilon(PRKCE)	GO:0002281~macrophage activation involved in immune response,GO:0006468~protein phosphorylation,GO:0006915~apoptotic process,GO:0007049~cell cycle,GO:0007155~cell adhesion,GO:0007165~signal transduction,GO:0007202~activation of phospholipase C activity,GO:0010634~positive regulation of epithelial cell migration,GO:0010763~positive regulation of fibroblast migration,GO:0010811~positive regulation of cell-substrate adhesion,GO:0018105~peptidyl-serine phosphorylation,GO:0030168~platelet activation,GO:0030838~positive regulation of actin filament polymerization,GO:0031397~negative regulation of protein ubiquitination,GO:0031663~lipopolysaccharide-mediated signaling pathway,GO:0032024~positive regulation of insulin secretion,GO:0032230~positive regulation of synaptic transmission,

		GABAergic,GO:0032467~positive regulation of cytokinesis,GO:0035556~intracellular signal transduction,GO:0035641~locomotory exploration behavior,GO:0035669~TRAM-dependent toll-like receptor 4 signaling pathway,GO:0038096~Fc-gamma receptor signaling pathway involved in phagocytosis,GO:0043123~positive regulation of I-kappaB kinase/NF-kappaB signaling,GO:0043278~response to morphine,GO:0043410~positive regulation of MAPK cascade,GO:0050730~regulation of peptidyl-tyrosine phosphorylation,GO:0050996~positive regulation of lipid catabolic process,GO:0051209~release of sequestered calcium ion into cytosol,GO:0051279~regulation of release of sequestered calcium ion into cytosol,GO:0051301~cell division,GO:0061178~regulation of insulin secretion involved in cellular response to glucose stimulus,GO:0070257~positive regulation of mucus secretion,GO:0071361~cellular response to ethanol,GO:0071380~cellular response to prostaglandin E stimulus,GO:0071456~cellular response to hypoxia,GO:0090303~positive regulation of wound healing,GO:2000273~positive regulation of receptor activity,GO:2000650~negative regulation of sodium ion transmembrane transporter activity,GO:2001031~positive regulation of cellular glucuronidation,
PPP3R1	protein phosphatase 3 regulatory subunit B, alpha(PPP3R1)	GO:0006470~protein dephosphorylation,GO:0007223~Wnt signaling pathway, calcium modulating pathway,GO:0033173~calcineurin-NFAT signaling cascade,GO:0038095~Fc-epsilon receptor signaling pathway,GO:0045944~positive regulation of transcription from RNA polymerase II promoter,GO:0051533~positive regulation of NFAT protein import into nucleus,GO:1900740~positive regulation of protein insertion into mitochondrial membrane involved in apoptotic signaling pathway,
PCMT1	protein-L-isoaspartate (D-aspartate) O-methyltransferase(PCMT1)	GO:0006479~protein methylation,GO:0030091~protein repair,GO:0098609~cell-cell adhesion,
RMND5A	required for meiotic nuclear division 5 homolog A(RMND5A)	GO:0042787~protein ubiquitination involved in ubiquitin-dependent protein catabolic process,GO:0043161~proteasome-mediated ubiquitin-dependent protein catabolic process,
RTN4	reticulon 4(RTN4)	GO:0006915~apoptotic process,GO:0007413~axonal fasciculation,GO:0021801~cerebral cortex radial glia guided migration,GO:0030308~negative regulation of cell growth,GO:0030334~regulation of cell migration,GO:0030517~negative regulation of axon extension,GO:0042981~regulation of apoptotic

		process,GO:0050771~negative regulation of axonogenesis,GO:0051292~nuclear pore complex assembly,GO:0060317~cardiac epithelial to mesenchymal transition,GO:0071786~endoplasmic reticulum tubular network organization,GO:0071787~endoplasmic reticulum tubular network assembly,GO:0098609~cell-cell adhesion,GO:2000172~regulation of branching morphogenesis of a nerve,
RECK	reversion inducing cysteine rich protein with kazal motifs(RECK)	GO:0001955~blood vessel maturation,GO:0007566~embryo implantation,GO:0030198~extracellular matrix organization,GO:0030336~negative regulation of cell migration,GO:0035115~embryonic forelimb morphogenesis,GO:1904684~negative regulation of metalloendopeptidase activity,
SEPTIN7	septin 7(SEPT7)	GO:0000910~cytokinesis,GO:0007067~mitotic nuclear division,GO:0016476~regulation of embryonic cell shape,GO:0051291~protein heterooligomerization,GO:0060271~cilium morphogenesis,GO:0098609~cell-cell adhesion,GO:1902857~positive regulation of nonmotile primary cilium assembly,
SGK3	serum/glucocorticoid regulated kinase family member 3(SGK3)	GO:0001558~regulation of cell growth,GO:0006468~protein phosphorylation,GO:0006883~cellular sodium ion homeostasis,GO:0006950~response to stress,GO:0010765~positive regulation of sodium ion transport,GO:0018105~peptidyl-serine phosphorylation,GO:0030334~regulation of cell migration,GO:0032411~positive regulation of transporter activity,GO:0034220~ion transmembrane transport,GO:0035556~intracellular signal transduction,GO:0042127~regulation of cell proliferation,GO:0042981~regulation of apoptotic process,GO:0051090~regulation of sequence-specific DNA binding transcription factor activity,
SESN2	sestrin 2(SESN2)	GO:0001932~regulation of protein phosphorylation,GO:0006635~fatty acid beta-oxidation,GO:0006914~autophagy,GO:0009749~response to glucose,GO:0016239~positive regulation of macroautophagy,GO:0030308~negative regulation of cell growth,GO:0030330~DNA damage response, signal transduction by p53 class mediator,GO:0032042~mitochondrial DNA metabolic process,GO:0032868~response to insulin,GO:0034599~cellular response to oxidative stress,GO:0036091~positive regulation of transcription from RNA polymerase II promoter in response to oxidative stress,GO:0043491~protein kinase B signaling,GO:0046323~glucose import,GO:0070328~triglyceride homeostasis,GO:0071230~cellular response to amino acid stimulus,GO:0071233~cellular response to leucine,GO:0072593~reactive oxygen

		species metabolic process,GO:0090526~regulation of gluconeogenesis involved in cellular glucose homeostasis,GO:0098869~cellular oxidant detoxification,GO:1900182~positive regulation of protein localization to nucleus,GO:1901031~regulation of response to reactive oxygen species,GO:1902010~negative regulation of translation in response to endoplasmic reticulum stress,GO:1904262~negative regulation of TORC1 signaling,GO:2000479~regulation of cAMP-dependent protein kinase activity,
SLC6A9	solute carrier family 6 member 9(SLC6A9)	GO:0003333~amino acid transmembrane transport,GO:0006810~transport,GO:0015816~glycine transport,GO:0036233~glycine import,GO:0060012~synaptic transmission, glycinergic,GO:0061537~glycine secretion, neurotransmission,
SNX4	sorting nexin 4(SNX4)	GO:0006897~endocytosis,GO:0015031~protein transport,GO:0016050~vesicle organization,GO:0032456~endocytic recycling,GO:1903595~positive regulation of histamine secretion by mast cell,
SPEN	spen family transcriptional repressor(SPEN)	GO:0000122~negative regulation of transcription from RNA polymerase II promoter,GO:0000398~mRNA splicing, via spliceosome,GO:0006351~transcription, DNA-templated,GO:0007219~Notch signaling pathway,GO:0016032~viral process,GO:0045892~negative regulation of transcription, DNA-templated,GO:0050769~positive regulation of neurogenesis,
SYPL1	synaptophysin like 1(SYPL1)	GO:0006810~transport,GO:0007268~chemical synaptic transmission,
THBS1	thrombospondin 1(THBS1)	GO:0000187~activation of MAPK activity,GO:0001666~response to hypoxia,GO:0001937~negative regulation of endothelial cell proliferation,GO:0001953~negative regulation of cell-matrix adhesion,GO:0002040~sprouting angiogenesis,GO:0002544~chronic inflammatory response,GO:0002576~platelet degranulation,GO:0002581~negative regulation of antigen processing and presentation of peptide or polysaccharide antigen via MHC class II,GO:0002605~negative regulation of dendritic cell antigen processing and presentation,GO:0006954~inflammatory response,GO:0006955~immune response,GO:0006986~response to unfolded protein,GO:0007050~cell cycle arrest,GO:0007155~cell adhesion,GO:0008284~positive regulation of cell proliferation,GO:0009612~response to mechanical stimulus,GO:0009749~response to glucose,GO:0010595~positive regulation of endothelial cell migration,GO:0010596~negative regulation of endothelial cell migration,GO:0010748~negative regulation of plasma membrane long-chain fatty acid transport,GO:0010751~negative regulation of nitric oxide mediated signal transduction,GO:0010754~negative regulation of cGMP-mediated

		signaling,GO:0010757~negative regulation of plasminogen activation,GO:0010759~positive regulation of macrophage chemotaxis,GO:0010763~positive regulation of fibroblast migration,GO:0016477~cell migration,GO:0016525~negative regulation of angiogenesis,GO:0018149~peptide cross-linking,GO:0030194~positive regulation of blood coagulation,GO:0030198~extracellular matrix organization,GO:0030335~positive regulation of cell migration,GO:0030511~positive regulation of transforming growth factor beta receptor signaling pathway,GO:0030823~regulation of cGMP metabolic process,GO:0032026~response to magnesium ion,GO:0032570~response to progesterone,GO:0032695~negative regulation of interleukin-12 production,GO:0032914~positive regulation of transforming growth factor beta1 production,GO:0033574~response to testosterone,GO:0034605~cellular response to heat,GO:0034976~response to endoplasmic reticulum stress,GO:0036066~protein O-linked fucosylation,GO:0040037~negative regulation of fibroblast growth factor receptor signaling pathway,GO:0042327~positive regulation of phosphorylation,GO:0042493~response to drug,GO:0042535~positive regulation of tumor necrosis factor biosynthetic process,GO:0043032~positive regulation of macrophage activation,GO:0043066~negative regulation of apoptotic process,GO:0043154~negative regulation of cysteine-type endopeptidase activity involved in apoptotic process,GO:0043536~positive regulation of blood vessel endothelial cell migration,GO:0043537~negative regulation of blood vessel endothelial cell migration,GO:0043652~engulfment of apoptotic cell,GO:0045727~positive regulation of translation,GO:0045766~positive regulation of angiogenesis,GO:0048266~behavioral response to pain,GO:0048661~positive regulation of smooth muscle cell proliferation,GO:0050921~positive regulation of chemotaxis,GO:0051592~response to calcium ion,GO:0051895~negative regulation of focal adhesion assembly,GO:0051897~positive regulation of protein kinase B signaling,GO:0051918~negative regulation of fibrinolysis,GO:0071356~cellular response to tumor necrosis factor,GO:0071363~cellular response to growth factor stimulus,GO:1902043~positive regulation of extrinsic apoptotic signaling pathway via death domain receptors,GO:2000353~positive regulation of endothelial cell apoptotic process,GO:2000379~positive regulation of reactive oxygen species metabolic process,GO:2001027~negative regulation of endothelial cell chemotaxis,GO:2001237~negative regulation of extrinsic apoptotic signaling

		pathway,
TOB1	transducer of ERBB2, 1(TOB1)	GO:0007184~SMAD protein import into nucleus,GO:0008285~negative regulation of cell proliferation,GO:0009967~positive regulation of signal transduction,GO:0017148~negative regulation of translation,GO:0030509~BMP signaling pathway,GO:0030514~negative regulation of BMP signaling pathway,GO:0045668~negative regulation of osteoblast differentiation,GO:0060212~negative regulation of nuclear-transcribed mRNA poly(A) tail shortening,GO:0060213~positive regulation of nuclear-transcribed mRNA poly(A) tail shortening,GO:1900153~positive regulation of nuclear-transcribed mRNA catabolic process, deadenylation-dependent decay,GO:1903507~negative regulation of nucleic acid-templated transcription,
TMEM245	transmembrane protein 245(TMEM245)	GO:0055085~transmembrane transport,
TNRC6A	trinucleotide repeat containing 6A(TNRC6A)	GO:0007223~Wnt signaling pathway, calcium modulating pathway,GO:0031047~gene silencing by RNA,GO:0035194~posttranscriptional gene silencing by RNA,GO:0035195~gene silencing by miRNA,GO:0035278~miRNA mediated inhibition of translation,GO:0048015~phosphatidylinositol-mediated signaling,
TRIM37	tripartite motif containing 37(TRIM37)	GO:0000122~negative regulation of transcription from RNA polymerase II promoter,GO:0006351~transcription, DNA-templated,GO:0032088~negative regulation of NF-kappaB transcription factor activity,GO:0035518~histone H2A monoubiquitination,GO:0036353~histone H2A-K119 monoubiquitination,GO:0046600~negative regulation of centriole replication,GO:0051091~positive regulation of sequence-specific DNA binding transcription factor activity,GO:0051092~positive regulation of NF-kappaB transcription factor activity,GO:0051865~protein autoubiquitination,GO:0070842~aggresome assembly,
YWHAG	tyrosine 3-monooxygenase/tryptophan 5-monooxygenase activation protein gamma(YWHAG)	GO:0000086~G2/M transition of mitotic cell cycle,GO:0006469~negative regulation of protein kinase activity,GO:0006605~protein targeting,GO:0009966~regulation of signal transduction,GO:0032869~cellular response to insulin stimulus,GO:0045664~regulation of neuron differentiation,GO:0048167~regulation of synaptic plasticity,GO:0061024~membrane organization,GO:0071901~negative regulation of protein serine/threonine kinase activity,GO:1900740~positive regulation of protein insertion into mitochondrial membrane involved in apoptotic signaling pathway,
VLDLR	very low density lipoprotein	GO:0000122~negative regulation of transcription from RNA polymerase II promoter,GO:0006869~lipid transport,GO:0006898~receptor-mediated

	receptor(VLDLR)	endocytosis,GO:0007165~signal transduction,GO:0007399~nervous system development,GO:0007613~memory,GO:0008203~cholesterol metabolic process,GO:0021517~ventral spinal cord development,GO:0034436~glycoprotein transport,GO:0034447~very-low-density lipoprotein particle clearance,GO:0038026~reelin-mediated signaling pathway,GO:0045860~positive regulation of protein kinase activity,GO:0048813~dendrite morphogenesis,GO:1900006~positive regulation of dendrite development,
ZFAND5	zinc finger AN1-type containing 5(ZFAND5)	GO:0001701~in utero embryonic development,GO:0001944~vasculature development,GO:0003016~respiratory system process,GO:0010761~fibroblast migration,GO:0048008~platelet-derived growth factor receptor signaling pathway,GO:0048705~skeletal system morphogenesis,GO:0048745~smooth muscle tissue development,GO:0060324~face development,
ZCCHC14	zinc finger CCHC-type containing 14(ZCCHC14)	
ZCCHC3	zinc finger CCHC-type containing 3(ZCCHC3)	
ZNF280B	zinc finger protein 280B(ZNF280B)	GO:0000122~negative regulation of transcription from RNA polymerase II promoter,GO:0006351~transcription, DNA-templated,GO:0007411~axon guidance,GO:0045892~negative regulation of transcription, DNA-templated,
