# Supplementary figures and images for: OncomiR miR-182-5p Enhances Radiosensitivity by Inhibiting the Radiation-Induced Antioxidant Effect through SESN2 in Head and Neck Cancer
Source: Antioxidants (Basel). 2021 Nov 14;10(11):1808. doi: 10.3390/antiox10111808 (PMC8614815; doi:10.3390/antiox10111808)

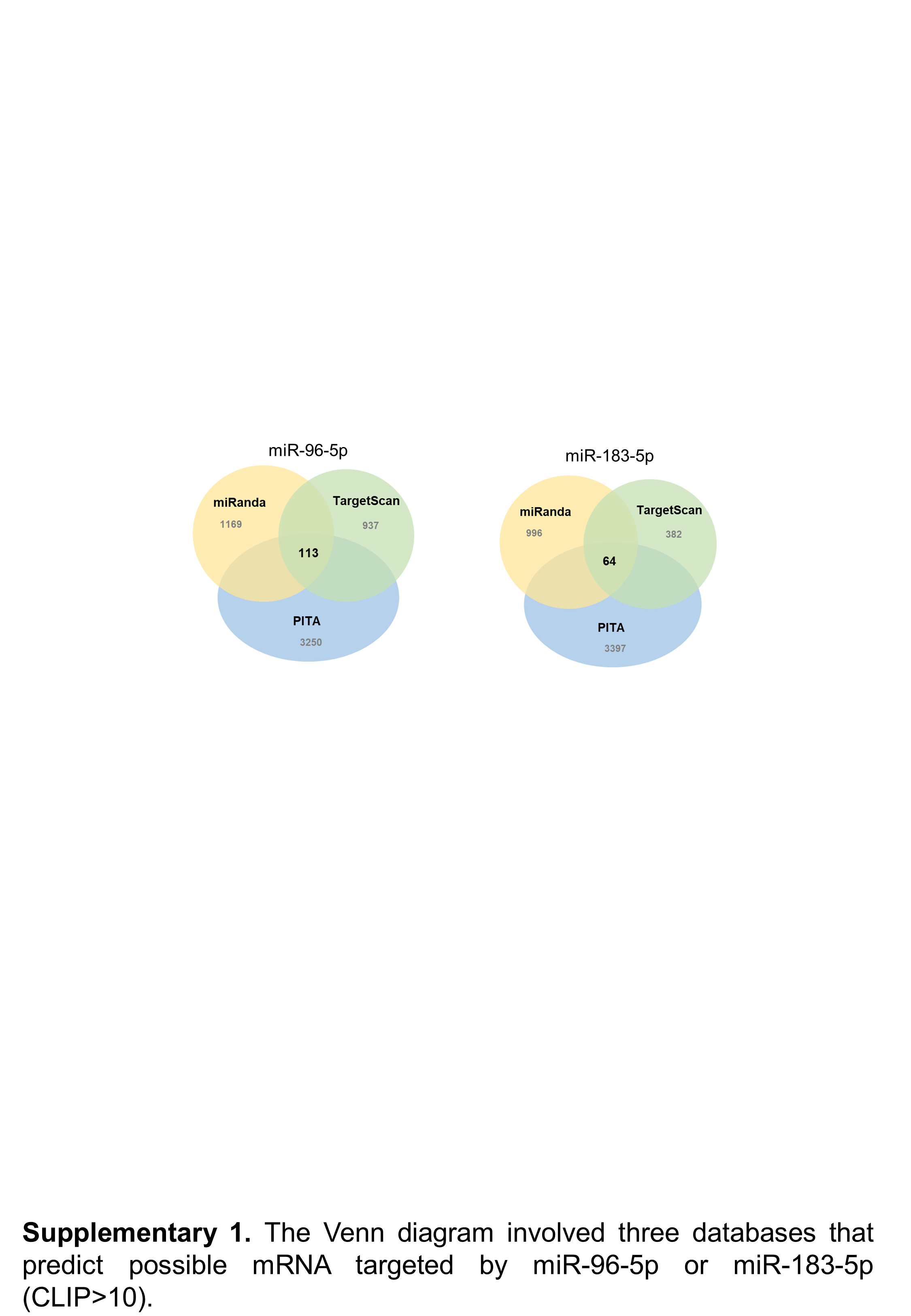

Supplement: Supplementary file 1 [file antioxidants-10-01808-s001.zip › Supplementary 1.TIF]

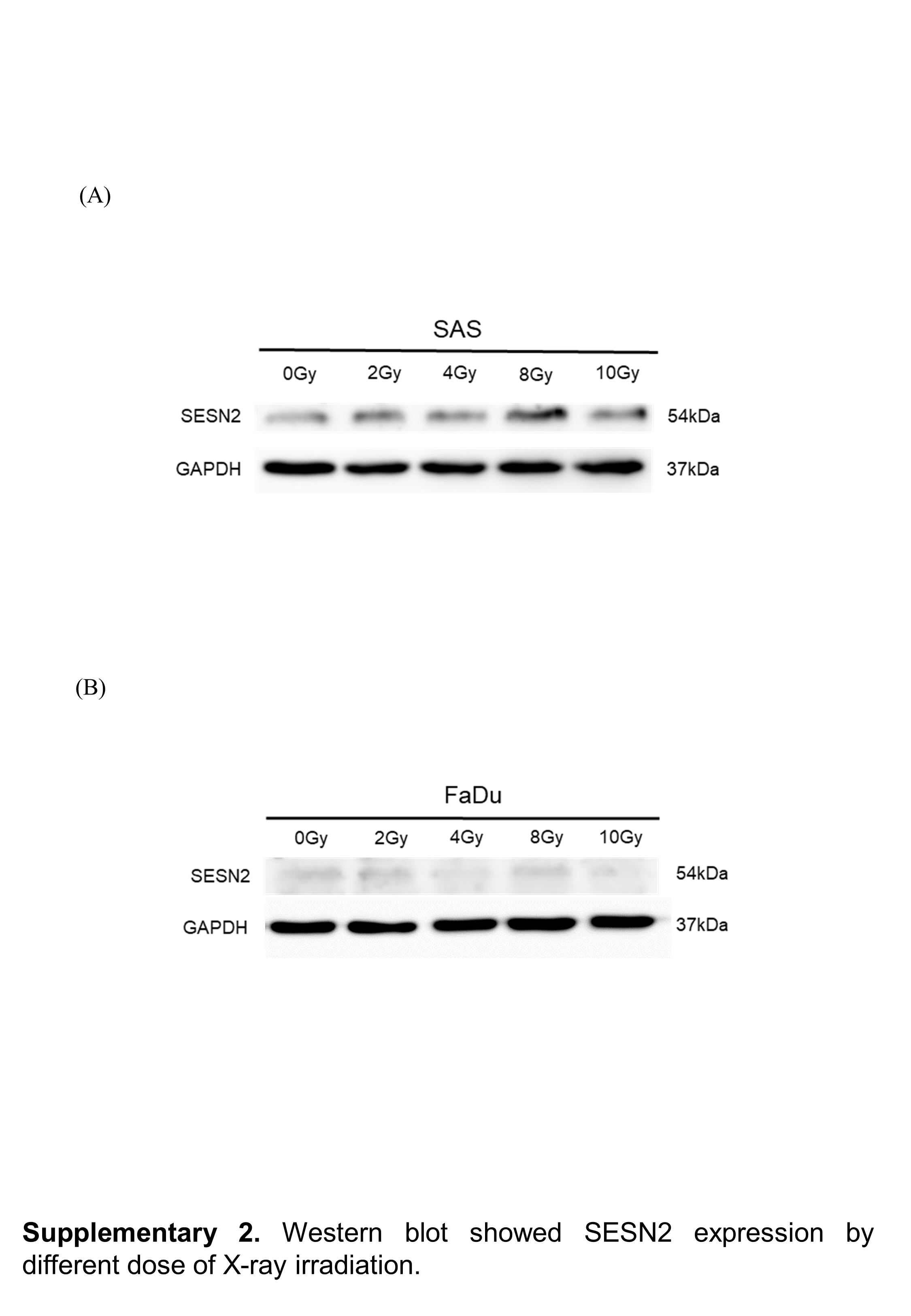

Supplement: Supplementary file 1 [file antioxidants-10-01808-s001.zip › Supplementary 2.TIF]

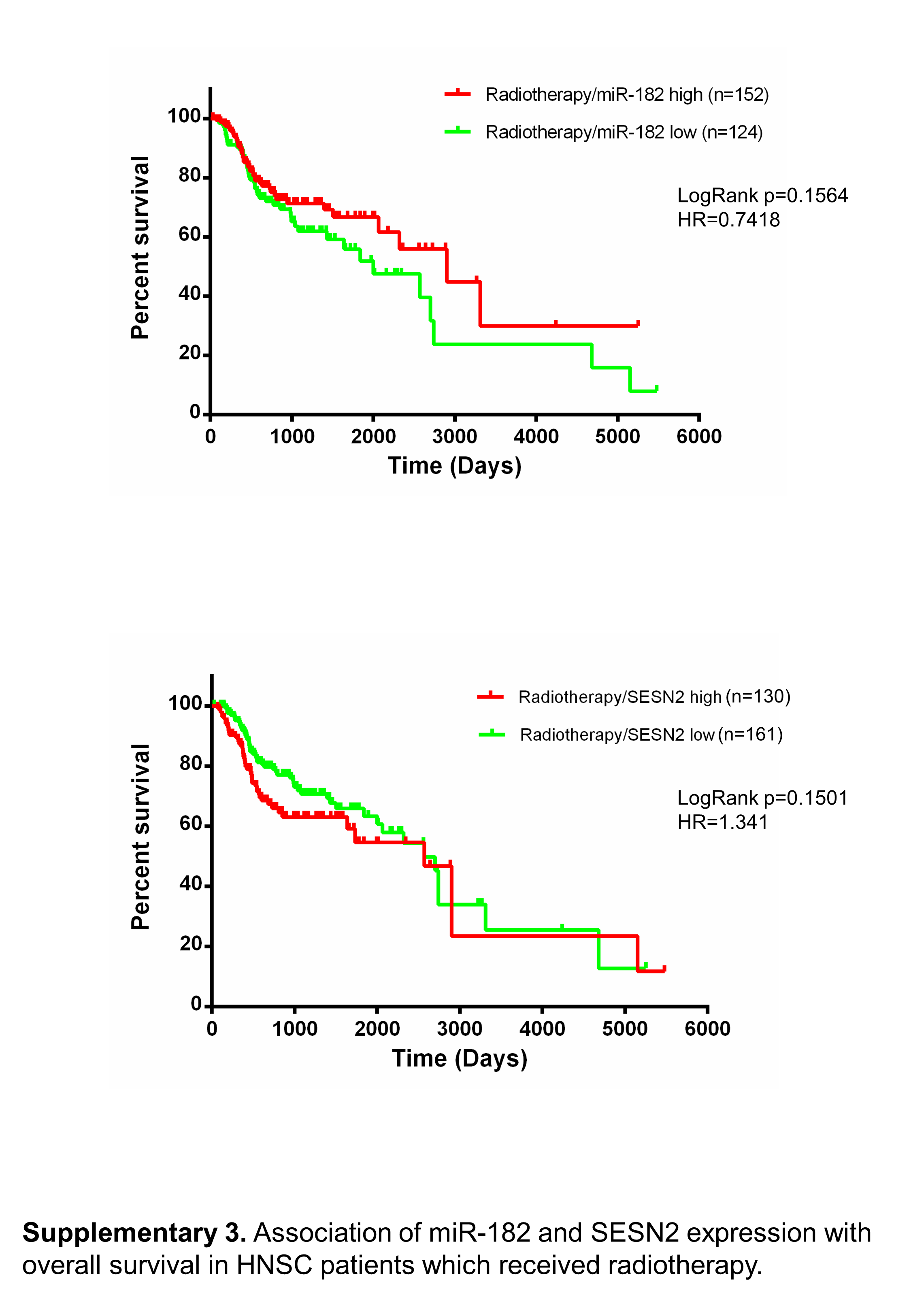

Supplement: Supplementary file 1 [file antioxidants-10-01808-s001.zip › Supplementary 3.TIF]
